# Direct-on-Filter FTIR Analysis of Respirable Crystalline Silica: A Field Study to Demonstrate Utility for Routine Non-regulatory Monitoring in Coal Mines

**DOI:** 10.1007/s42461-024-01154-4

**Published:** 2024-12-05

**Authors:** Garek Elie, Rohit Pandey, Emily Allyn Sarver

**Affiliations:** Mining and Minerals Engineering, Virginia Tech, Blacksburg, USA

**Keywords:** Respirable crystalline silica, Coal mine dust, End-of-shift silica analysis, CPDM, Portable FTIR

## Abstract

Crystalline silica is generally considered the most hazardous constituent of respirable coal mine dust, and new federal rules are aimed at further limiting exposures in US mines. For regulatory purposes, respirable crystalline silica (RCS) is monitored by collecting filter samples, which are analyzed for quartz (the predominant form of RCS) by Fourier transform infrared spectroscopy (FTIR) in a centralized laboratory. To enable more timely results, NIOSH has developed a direct-on-filter (DOF) FTIR analysis method and free “FAST” software that can be used in the field. For samples collected under specific conditions, *FAST* processes the FTIR spectral data to determine quartz mass (and mass concentration with user inputted sampling duration and flow rate). The DOF FTIR method is not approved for regulatory RCS monitoring; however, it could be quite useful for routine non-regulatory monitoring. This field study demonstrates how the method (including *FAST*) could be applied for that purpose, specifically using low-cost and widely available sampling components (i.e., generic 3-piece styrene cassettes and an expandable cyclone-cassette holder assembly that is designed to be compatible with these cassettes). Moreover, to demonstrate how quartz percentage could be estimated in the field, sampling for this study included continuous personal dust monitors (CPDMs), which are designed to measure respirable dust mass concentration. While the DOF FTIR method enables field-measurement of quartz mass (and mass concentration) using a filter sample, field-measurement of the respirable dust mass (and mass concentration) on that filter sample is impractical. This would require determination of the total sample mass, which is typically done with a sensitive microbalance. Thus, the paired CPDM approach is presented workable solution.

**Supplementary Information:**

The online version contains supplementary material available at https://doi.org/10.1007/s42461-024-01154-4.

## Introduction

Respirable crystalline silica (RCS) is a serious yet ubiquitous hazard in many occupational environments. In coal mines, RCS is one of many constituents of the total respirable coal mine dust (RCMD) [1], and it predominantly present as α-quartz (termed “quartz” from here) [2]. RCS has been linked to resurgence of disease in US coal mine workers [3, 4]. Federal monitoring data suggests RCS levels have been gradually declining in coal mines [5], especially since the US Mine Safety and Health Administration (MSHA) reduced the personal exposure limit for RCMD and mandated new monitoring technology as part of a 2014 rule [19]. However, to better protect workers in all US mining sectors, MSHA recently published a “new silica rule” which lowers the personal exposure limit [5]. The new limit is 50 μg/m^3^ for a full work shift, calculated as an 8-h time-weighted average (TWA).

Currently, RCS is monitored in US coal mines by collecting RCMD filter samples and sending them to a certified lab for analysis of quartz, and this will continue to be the approach for regulatory monitoring under MSHA’s “new silica rule.” Briefly, the procedure can be summarized in the following steps: First, the filter (37-mm polyvinyl chloride, PVC, with nominal 5 μm pore size) is pre-weighed. This requires a sensitive microbalance in the laboratory, and in most cases pre-weights are measured by the filter vendor, prior to loading the filter (and filter support) into the sampling cassette. Then, the RCMD sample is collected in field using an air pump with a cyclone pre-selector (to remove oversized particles) to pull the respirable dust directly onto the filter housed within a closed cassette. The sampling time and flow rate are recorded, such that the total volume of air sampled can be determined. Next, the sample is sent to a lab for analysis. This includes post-weighing the filter to enable determination of total RCMD sample mass and then analysis by a standard Fourier transform infrared (FTIR) method to determine quartz mass. Using the quartz mass and sampled air volume, the quartz concentration can be computed, and the same can be done to determine RCMD mass concentration. (Notably, the quartz and RCMD mass have historically been reported on the basis of “MRE equivalency,” as explained below.) Finally, using the ratio of the quartz and RCMD concentrations, the quartz percentage can be computed.

While the general procedure for coal mine RCS sampling and analysis will not change as the “new silica rule” comes into enforcement (in 2025), it is important to note two specific details that will change. First, there will be more flexibility in the sampler type. Heretofore, a “coal mine dust personal sampling unit” (CMDPSU) has been required, which specifically uses a 10-mm nylon cyclone (Dorr-Oliver type) at a flow rate of 2.0 L/min. Under the new regulations, any sampler that is compliant with ISO 7708 for respirable dust is acceptable, and there are numerous samplers (with different cyclone and flow rate combinations) that meet this requirement. Second, quartz mass will no longer be determined on the basis of MRE equivalency. This convention stems from the fact that coal mine dust regulations in the US were historically based on data derived from sampling with an MRE sampler (i.e., which was designed by the Mining Research Establishment). The MRE sampler collects a slightly different size fraction of dust than the CMDPSU (at 2.0 L/min), so CMDPSU measurements of both quartz and RCMD have been corrected to be MRE-equivalent using a constant 1.38 multiplier (see [8]).

As noted, analysis of quartz mass in RCMD samples typically done using lab-based FTIR methods. These include the standard MSHA Method P7 [6], which has been used for regulatory compliance sampling in the US, and the analogous NIOSH Method 7603 [7]. Both require careful preparation of the sample prior to the FTIR analysis, which involves ashing the sample to remove the filter and combustible material, and then redeposition of the residue for the FTIR analysis. In all, the time from sampling to results may be days to weeks. Not only is this unfavorable for timely correction of overexposures indicated by personal samples, but it is also unfavorable for routine monitoring to support engineering work (e.g., to track RCS trends with mining conditions, evaluate controls) Moreover, the standard analysis is relatively expensive. The net result is that, at present, RCS monitoring is typically limited to that which is required for regulatory compliance. However, especially considering the implications of the “new silica rule,” there is both a need and desire to do more.

### RCS Analysis by Direct-on-Filter FTIR

While real-time RCS monitoring would be ideal, there are currently no proven technologies for this in coal mines [9] (National Academies Press, 2018). However, NIOSH has developed and standardized a method for direct-on-filter (DOF) spectroscopy FTIR analysis [10–13]. It uses the same type of filter sample that would be collected for the lab-based analysis described above. But, if the DOF method is done with a portable FTIR spectrometer, it can enable rapid and field-based analysis. Further, NIOSH has developed a free software called the *Field Analysis of Silica Tool* (*FAST*) to simplify data processing [14]. In essence, *FAST* takes the user-inputted FTIR spectrum and sampling conditions (i.e., target flow rate, cyclone/cassette combination) and outputs the computed quartz mass. With user-inputted sampling time and flow rate, the quartz mass concentration is also output. However, since weighing the sample filter in the field is not practical (i.e., a microbalance is not typically available), the quartz percentage cannot be directly determined.

Importantly, for DOF analysis, sampling conditions are critical for translating the FTIR spectral results to quartz mass. To explain: the analysis is conducted on a small, central area of the filter, but the result must be extrapolated across the entire filter area [11–13, 15, 16]. The extrapolation model is dependent on the expected dust deposition pattern, which is dependent on the geometry of the cassette and inlet airflow conditions (i.e., sampling flow rate and the effective inlet diameter, which depends on how the cassette is connected to the cyclone pre-selector). Thus, for accurate quartz measurements using the DOF FTIR method, the extrapolation model must be specific to the sampling conditions.

**Fig. 1:**
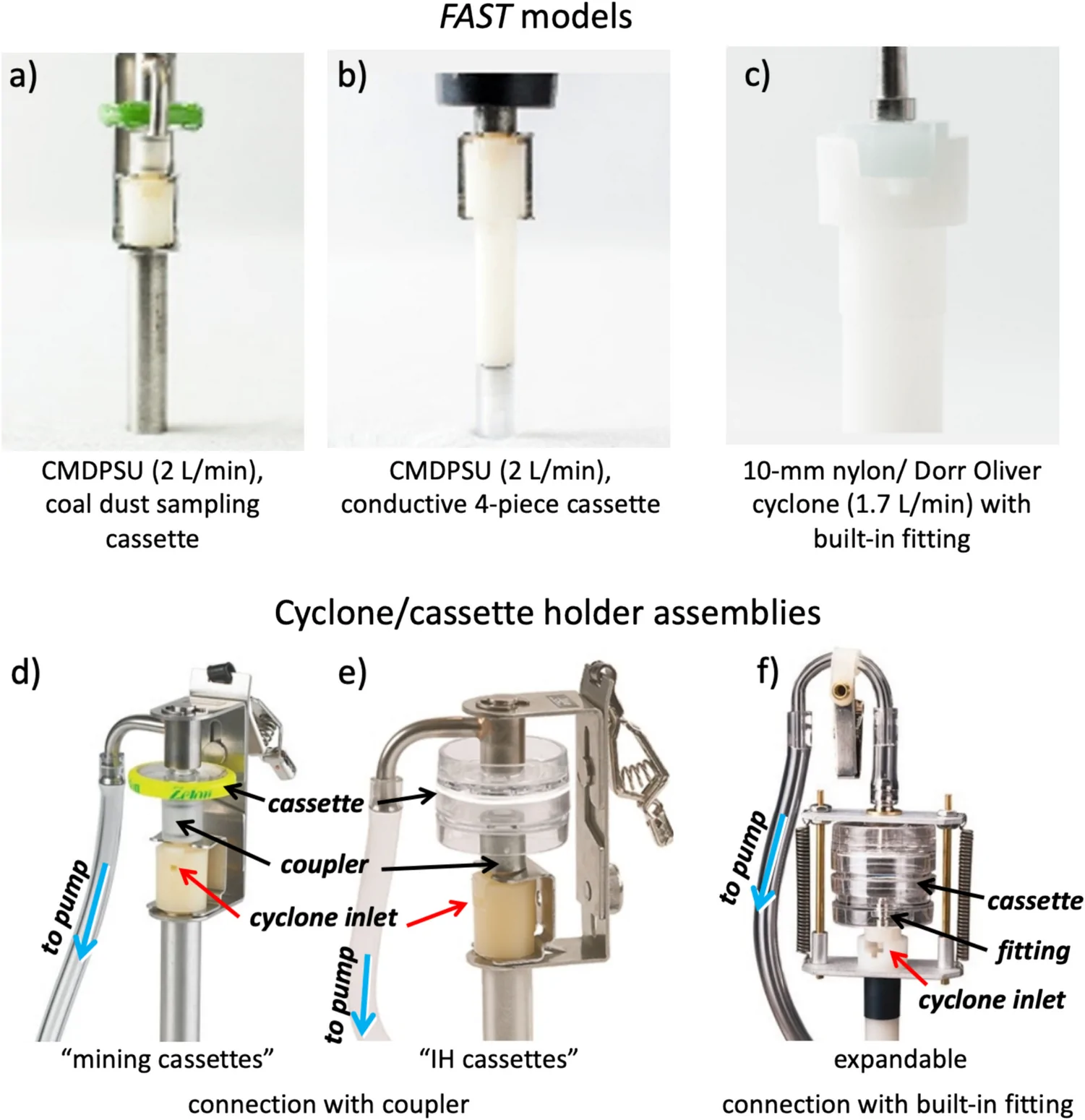
Example respirable mine dust sampling cyclone and cassette combinations; all combinations shown use a 10-mm Dorr-Oliver cyclone, but they differ in terms of the cassette type, cyclone-cassette assembly, and flow rate. The top row shows combinations that have been modeled in NIOSH’s Field Analysis of Silica Tool (*FAST*) with images taken as screenshots from the FAST user interface. The bottom row shows standard cyclone-cassette assemblies with images taken from Zefon.com and annotated. For coal mine dust (sampled at 2.0 L/min), the *FAST* models assume a cyclone-cassette assembly that uses a coupler: model **a** is for a “coal dust sampling cassette,” which would be sampled using the assembly shown in **d**. Model **b** is for a special 4-piece conductive cassette, which presumably would be sampled with a modification of the assembly shown in e) (i.e., per photos in [17], which details design of the cassette, it appears the top of the holder assembly would be removed to accept the height of the 4-piece cassette). *FAST* also includes model **c** for a cyclone-cassette assembly with a built-in fitting instead of the coupler; this assembly can accept a 3- or 4-piece cassette; however, the assumed sampling flow rate is 1.7 L/min (note: the *FAST*-output quartz mass is MRE-equivalent for models **a** and **b**, but not for **c**)

With an eye toward eventual capabilities for end-of-shift personal RCS monitoring in coal mines, (prior to the “new silica rule”) NIOSH developed and included in *FAST* two models for RCMD samples collected with CMDPSUs (Fig. 1). One is for use with traditional coal mine sampling cassettes (Fig. 1a, sometimes called “MSA” cassettes due to the historical vendor), which are the type that has been used by MSHA for compliance RCS sampling. The other model is for use with a special 4-piece conductive cassette (Fig. 1b), which NIOSH specifically designed for the DOF FTIR analysis [17]. Notably, both models currently include the MRE-equivalency conversion and a sampling flow rate of 2.0 L/min (i.e., pre- “new silica rule” sampling conditions), and both assume the cassette was connected to the cyclone using a holder assembly that includes a coupler. (This is sometimes referred to as the “mining” or “IH” style holder assembly as shown in Fig. 1d and Fig. 1e, respectively. )

Pampena et al. (2020) field tested the DOF FTIR method with the coal mine sampling cassettes and found variable accuracy (against MSHA Method P7 as reference) [18]. This was attributed to variable dust deposition pattern on the sample filter and was consistent with earlier findings by Miller et al. (2013) [15]. On the other hand, the 4-piece cassette yields a more reproducible deposition pattern, with heavier loading in the filter center that gradually dissipates radially toward the filter edge [17]. This pattern enables a more accurate extrapolation model. Further, the 4-piece design of the cassette enables the DOF FTIR analysis with minimal sample handling due to two main features. First, the filter support is a stainless-steel ring, which is fully open in the center to allow IR beam transmission. Second, the filter and support are sandwiched between the middle two pieces of the cassette; for analysis, the top and bottom (outer) pieces can simply be removed, and the sandwich can be placed in a cradle to align the filter center with the IR beam.

### Considerations for Routine Non-regulatory Monitoring

While the DOF FTIR method has primarily been considered in the context of personal RCS exposure monitoring, application for environmental monitoring is a more likely entry point for coal mines. To gain experience and confidence with this new capability, mines might want to use it for routine non-regulatory monitoring, for example, in fixed or standardized locations to track RCS trends with operational or geological conditions, or to evaluate dust controls. For these sorts of applications, data utility and costs are obvious considerations.

With respect to data, one issue could be the inability to directly determine quartz percentage using the DOF FTIR method. As noted, this metric is readily determined with standard lab analysis of RCMD filter samples because the filter is weighed prior to preparation for the quartz analysis by FTIR. Thus, most coal mines have considerable knowledge of the typical quartz percentages in their RCMD, and this data could be quite useful for a range of engineering purposes (e.g., to evaluate RCS generation from various geological features). However, the DOF FTIR method only determines quartz mass (and mass concentration if the sampled air volume is known). While it is impractical to weigh the filter sample in the field to determine the total RCMD mass, an alternative approach could be to estimate the RCMD mass by pairing the CMDPSU with a continuous personal dust monitor (CPDM) during sampling. The CPDM was developed and is now mandated, for RCMD monitoring in US coal mines [9, 19]. In essence, it tracks (MRE-equivalent) mass concentration on 1-min intervals over its entire sampling period. Thus, if a CPDM is paired with the CMDPSU, the quartz percentage could be estimated using the quartz concentration derived from DOF FTIR analysis of the filter sample and the CPDM’s RCMD concentration.

Regarding costs, adoption of the DOF FTIR method represents an investment. In terms of equipment, a portable FTIR spectrometer is needed, though operators with multiple mines might consider a single instrument that could be shared across sites. Other equipment costs should be limited to filter samplers and perhaps related calibration accessories. Notably, some mines may still own CMDPSUs since these were required for RCMD compliance monitoring prior to implementation of the CPDM in 2016 [9]. For consumables, sampling supplies (i.e., cassettes, filters and filter supports) are expected to be the main costs. While the special 4-piece conductive cassettes have been designed to minimize sample handling, mines could opt to use generic 3-piece styrene cassettes for routine non-regulatory monitoring. This option would require sample handling since the filter cannot be analyzed while still inside the cassette. However, at the time of publication, 3-piece cassettes were about seven times less expensive than the 4-piece cassettes.^1^ Notably, the 3-piece cassettes were the basis of NIOSH’s early work to develop the DOF FTIR method (e.g., see [15]). They have essentially the same deposition pattern (and pattern reproducibility) as the special 4-piece cassette [17], so long as the same flow rate and cyclone-cassette holder assembly is used.

Indeed, the cyclone-cassette holder type is a critical parameter for the DOF FTIR data analysis. As mentioned, the *FAST* model for use with 4-piece cassettes assumes that sampling was conducted with a CMDPSU using an IH style holder with a coupler to connect the cyclone and cassette (Fig. 1e); the coupler is like a sleeve that fits around the outside diameter of the cyclone outlet and cassette inlet, thereby enabling airflow through the full diameter of the cassette inlet tube. However, this particular cyclone-cassette holder, as designed, is not compatible with the 4-piece cassette—and due to the tolerance on some holders can be challenging to use with a 3-piece cassette. This is because, as shown in Fig. 1e (with a 2-piece cassette), the holder uses a metal frame to secure the cyclone-cassette connection. However, the height of the 4-piece cassette is too tall to fit within the frame. Thus, the assembly would either require modification or the cassette would be unsecured (i.e., with top portion of frame removed).

An alternative option is shown in Fig. 1f, which is an assembly with a spring-enabled expandable frame that can easily fit the 4- or 3-piece cassette to secure it. As designed though, this holder assembly uses a built-in fitting to connect the cyclone outlet to the cassette inlet. Because the fitting goes inside the cassette inlet tube, it effectively reduces the airflow diameter and thereby increases the velocity, which can alter the dust deposition pattern on the sample filter. Importantly, *FAST* does include a model to cover this combination of cassette and cyclone-cassette assembly, but it assumes a sampling flow rate of 1.7 L/min (see Fig. 1c). These conditions are consistent with respirable sampling for metal/nonmetal mines and should also be acceptable for RCS monitoring in coal mines under the “new silica rule” since they are ISO 7708 compliant. However, *FAST* does not include a model to cover the 4- or 3-pc cassette with built-in fitting from the cyclone to cassette at 2.0 L/min. While the 2.0 L/min flow rate is not expected to be used for compliance sampling moving forward, sampling at this flow rate could still be important in certain instances. For example, given that all prior data on quartz concentrations (and percentages) were collected at 2.0 L/min, there will undoubtedly be keen interest in understanding the effect of reduced flow rate on quartz measurements. (The flow rate controls the cut size of the cyclone and, depending on the specific particle size distribution of different dust constituents, quartz mass concentration and percentage could vary somewhat with flow rate.) Moreover, considering the aforementioned strategy to pair CMDPSUs and CPDMs for sampling to estimate quartz percentage, use of the 2.0 L/min flow rate on the CMDPSU would be needed since the CPDM was essentially designed to match this condition on the basis of RCMD concentration [20].

Based on the motivations described above, the aim of this field study was to demonstrate the DOF FTIR method for RCS monitoring in coal mines, specifically using (a) low-cost yet practical consumables (i.e., pre-loaded 3-piece styrene cassettes), (b) cassette-cyclone holder assemblies that can be used off-the-shelf, as-designed (i.e., the expandable type shown in Fig. 1e), and (c) paired CPDM instruments to enable estimation of quartz percentage (in addition to quartz mass).

## Methods and Materials

### Mine Sampling

This field study was conducted in an underground room and pillar mine (called “Mine 28” from here) located in the Illinois coal basin. At the time of sampling, Mine 28 was producing coal from the Indiana V seam. Sampling was conducted in two different sections (MMU1 and MMU3), both of which had 11 entries and operated with a split air, blowing ventilation design. In general, the roof strata in MMU1 consisted of highly fractured shale, whereas the roof in MMU3 was a structurally stronger limestone.

As shown in Table 1, dust sampling was conducted during 10 separate events (i.e., sampling in a particular MMU on a particular day). For each event, relative heights of coal and roof rock being mined in the section were recorded, and airflow quantities were estimated in each sampling location based on airway dimensions and velocities measured using a 4-inch anemometer (see Table S1 in the Supplemental Information). Production was at typical levels for Mine 28 during all events (i.e., there was not significant downtime of equipment) .

**Table 1:** Summary of dust sampling in Mine 28

Event	MMU	Sampling locations	Sampling duration (min)	Average mining height (ft)	
				**Coal**	**Rock**
E1	1	L1, L2, L3, L4	288	4.58	2.23
E2	1	L1, L2, L3, L4	311	4.79	2.00
E3	3	L1, L3	308	7.58	1.04
E4	3	L2, l4	288	7.67	0.33
E5	3	L2, L3	255	8.13	0.38
E6	1	L1, L2	289	4.79	1.88
E7	3	L1, L2	292	8.33	0.04
E8	3	L2, L3	262	7.75	0.42
E9	1	L1, L2	259	6.00	0.50
E10	3	L1, L2	325	7.38	0.54

**Fig. 2:**
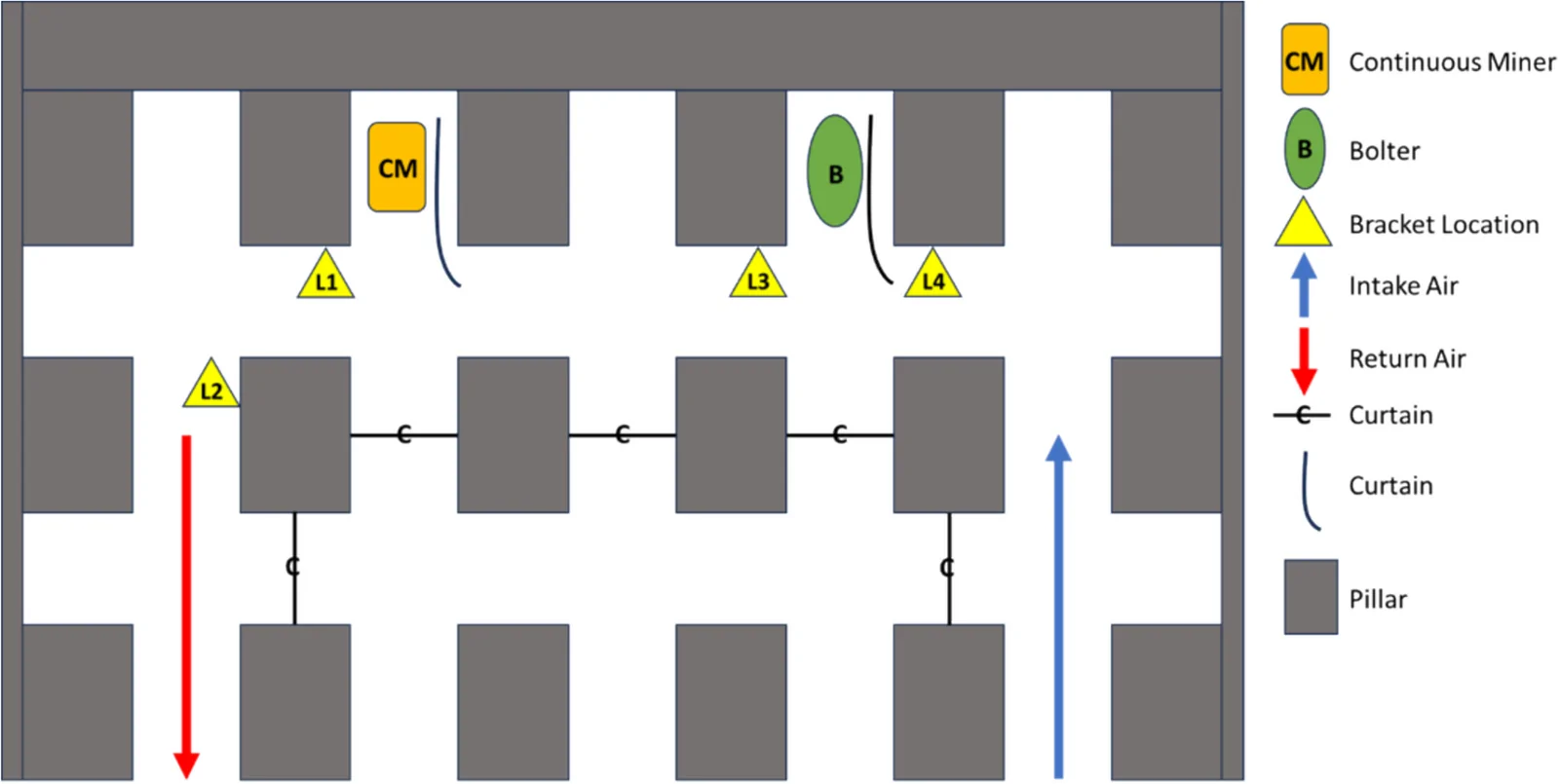
General locations where each sample “set” was collected

During events 1 and 2, samples were simultaneously collected in the four standardized locations shown in Fig. 2: location 1 was in the return of the continuous miner, location 2 was in the section return (last open crosscut in the return entry), location 3 was in the return of the roof bolter (upwind the continuous miner), and location 4 was in the section intake. During the other events, samples were only collected in two of the four locations (see Table 1).

**Fig. 3:**
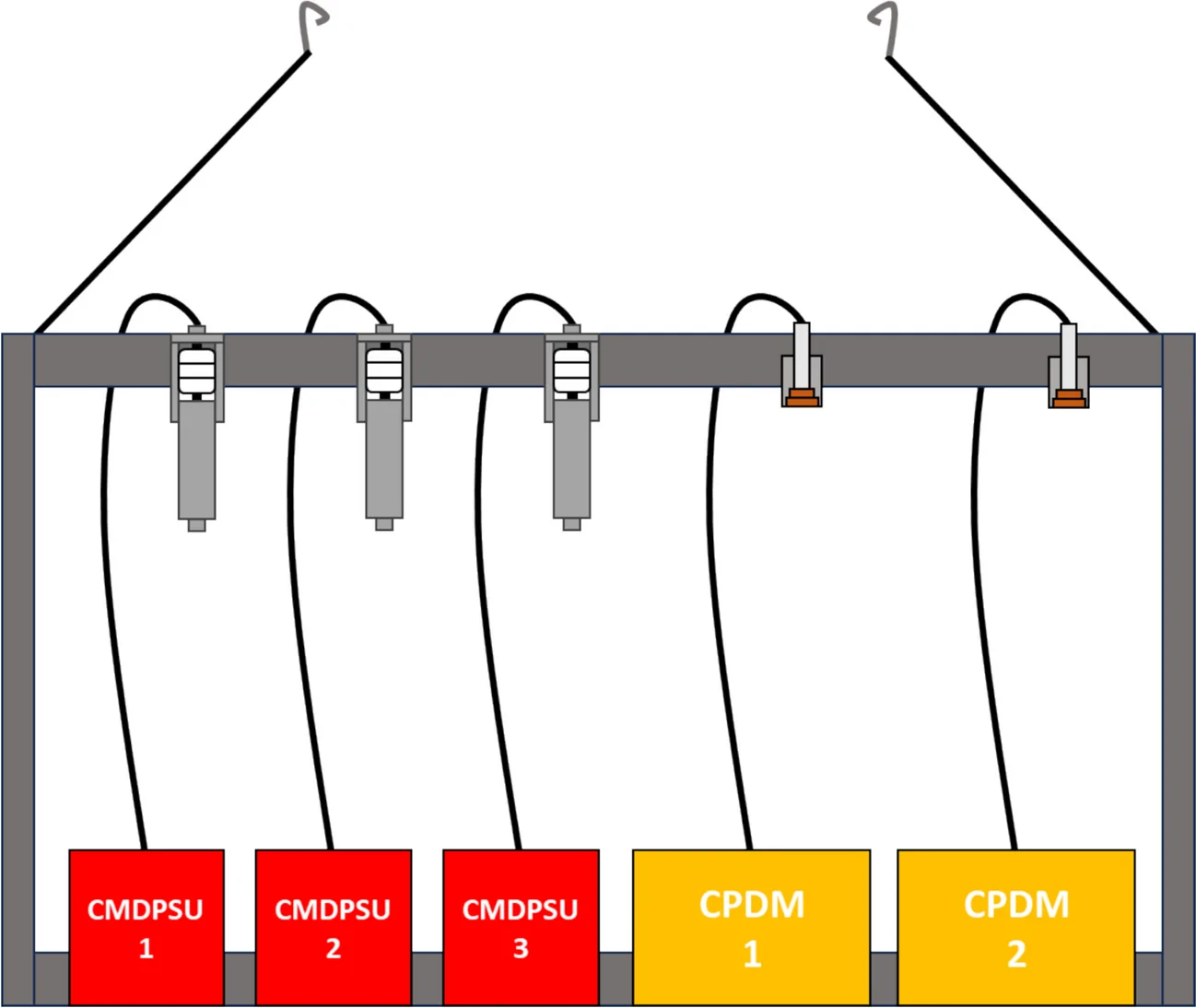
Schematic of equipment used to collect a “set” of RCMD samples (i.e., each event × location)

In each event, a “set” of RCMD filter samples were collected in each location; across all events and locations (Table 1), there were a total of 24 sets. Each set included three filter samples (triplicates) collected with CMDPSUs (72 samples total). Additionally, paired with the CMDPSUs in each set were either one (events 1–2) or two CPDMs (events 3–10); the only exception was event 2 in location 3, for which no CPDM was available. In all, this yielded a total of 39 CPDM data logs (i.e., one log per CPDM in each unique sampling event and location). All sampling equipment was attached to an angled-steel frame, such that the air inlets were oriented in the same direction (Fig. 3). Within each sample set, the CMDPSUs were switched on and off simultaneously, and the start and stop times were recorded to determine the total sampling duration. However, the CPDM has a substantial warm-up time (typically about 35 min), so these instruments were turned on prior to being transported into the mine.

Each CMDPSU consisted of an Escort ELF air pump operating at 2.0 L/min, a 10-mm nylon Dorr-Oliver cyclone, and a 3-piece styrene cassette loaded with a pre-weighed 37-mm PVC filter and cellulose support pad. The cyclone-cassette assembly was of expandable type shown in Fig. 1f. (All CMDPSU parts were purchased from Zefon International, Ocala, FL.) Pump flow rates were checked and calibrated prior to each event using a Gilian Gilibrator-2 (Sensidyne, St. Petersburg, FL). The CMDPSU filter samples were analyzed gravimetrically to determine RCMD mass and TWA RCDM concentration, and by FTIR to determine quartz mass and TWA quartz concentration (see below).

The CPDMs were Thermo Scientific PDM3700 Personal Dust Monitors, operated at 2.2 L/min with a Higgins-Dewell cyclone (which has a similar penetration efficiency to the Dorr-Oliver cyclone at 2.0 L/min per [20]). Each monitor was cleaned and calibrated prior to each sampling event. The CPDM collects a dust sample on a special filter stub, which is attached to a tapered element oscillating microbalance (TEOM). As dust accumulates on the filter, the TEOM tracks changes in oscillation frequency and uses a calibration factor to determine the MRE-equivalent dust mass deposited on a 1-min time interval (logged in the instrument’s data file); under typical use, the instrument uses the mass accumulation to continuously compute and display mass concentration (i.e., as a rolling 15-min average or the current full-shift equivalent). For this study, the CPDM’s data log was downloaded following each sampling event and the sample mass accumulated during the specific time interval corresponding to the paired CMDPSUs was used to derive an estimate of the TWA RCMD concentration during this time. This concentration was considered as an alternate to the value that can be determined from the CMDPSU filters gravimetrically—since such gravimetric analysis is impractical in the field.

In both the mine sections studied, bulk samples of primary dust source materials were also collected. The bulk samples included large pieces of raw coal (MMU1 and MMU3) and roof rock (shale, MMU1), which were hand-picked from the production belt, plus a sample of the limestone rock dusting product the mine uses. Since very little roof rock was being cut in MMU3 during the study, a sample of the material from the roof bolter dust collection system was taken to represent dust generated from the roof in this section. Each material was used to generate respirable dust samples (triplicates) in the laboratory to gain insights to the quartz content. Briefly, this was done by aerosolizing a small mass of each source material within a small enclosure and collecting the respirable dust using the same CMDPSU apparatus used for the mine sampling. (For these samples, the PVC filters were placed in 2-piece rather than 3-piece cassettes.) The filters were pre- and post-weighed to determine the sample mass. Notably, while the roof bolter material and the rock dust product were already very fine, the raw coal and rock materials were pulverized in the lab and sieved (to less than 65 μm) before generating the respirable dust samples.

### Gravimetric Analysis

Filter samples collected with CMDPSUs were pre-weighed and post-weighed in the authors’ Virginia Tech laboratory using a Cubis MSE6.6S microbalance (Sartorius, Gottingen, Germany) which has a readability of 2 μg. For each sample, the MRE-equivalent RCMD mass (mg) was determined using Eq. (1), and the TWA concentration (mg/m^3^) was determined using Eq. (2):

1 $${M}_{RCMD\_filter}\mathrm{(mg)}=1.38\times \left({F}_{2}-{F}_{1}\right)\mathrm{(mg)}$$

2 $${C}_{RCMD\_filter}\text{ (}\frac{\mathrm{mg}}{{\mathrm{m}}^{3}}\mathrm{)}=\frac{{M}_{RCMD\_filter} \mathrm{(mg)}}{2.0 \left(\frac{\mathrm{L}}{{\mathrm{min}}}\right) \times t \mathrm{(min)}}\times \frac{1000 {\mathrm{L}}}{{\mathrm{m}}^{3}}$$

where *F*_*1*_ and *F*_*2*_ are the filter pre- and post-weight (mg), respectively; 1.38 is the constant MRE-equivalency factor; *t* is the sampling duration (min); and 2.0 L/min is the sampling flow rate for the CMDPSU.

To derive estimated RCMD mass (mg) and TWA concentration (mg/m^3^) values from the CPDM data, Eqs. (3) and (4), respectively, were used:

3 $${M}_{RCMD\_CPDM}\text{ (mg)}=\left({S}_{2}-{S}_{1}\right) \mathrm{(mg)}$$

4 $${C}_{RCMD\_CPDM}\text{ (}\frac{\mathrm{mg}}{{\mathrm{m}}^{3}}\mathrm{)}=\frac{{M}_{RCMD\_CPDM} \mathrm{(mg)}}{2.2 \left(\frac{\mathrm{L}}{{\mathrm{min}}}\right) \times t \mathrm{(min)}}\times \frac{1000 {\mathrm{L}}}{{\mathrm{m}}^{3}}$$

where *S*_*1*_ and *S*_*2*_ are the CPDM’s recorded values for filter stub mass (mg) at the start time and stop time of the paired CMDPSU samplers, respectively; *t* is again the sampling duration (min) of the CMDPSU samplers; and 2.2 L/min is the sampling flow rate for the CPDM (L/min). (Note the 1.38 MRE-equivalency factor is not needed in Eq. 3 because the CPDM is programmed to record MRE-equivalent mass.)

For the respirable dust samples that were lab-generated from the dust source materials collected in Mine 28, the filters were pre- and post-weighed (using the same microbalance as above). The sample mass (mg) was determined by simple difference using Eq. (5):

5 $${M}_{RD}\mathrm{(mg)}=\left({F}_{2}-{F}_{1}\right) \mathrm{(mg)}$$

### DOF FTIR Analysis

The PVC filter samples collected with CMDPSUs were analyzed by DOF FTIR using an Alpha II Compact spectrometer and OPUS software (Bruker Optics, Billerica, MA), and then, the data was inputted into NIOSH’s *FAST* software. To scan each filter, it needed to be transferred from its respective 3-piece sampling cassette to the “sandwich” made by the two middle pieces of a 4-piece cassette (with steel ring support). For this, clean stainless-steel tweezers were used. Then, the sandwich was placed into a 3D-printed cradle and stand holder (design files freely available from NIOSH, https://3d.nih.gov/entries/3dpx-013209), which are positioned inside the spectrometer’s analysis compartment to align with the IR beam.

Analysis with the FTIR instrument was conducted following the method outlined by NIOSH [11]. Briefly, the center (6-mm diameter area) of each sample filter was scanned to measure the absorbance spectrum between 4000^ cm−1^ and 400 cm^−1^ [11]; a blank PVC filter was also scanned. Specifically, the instrument was set to obtain 16 consecutive scans using Blackman-Harris 3-term apodization with a 4 cm^−1^ resolution. Using a NIOSH-developed macro for the OPUS software, distortions were removed from the spectrum with a “rubber band baseline correction” that includes 64 baseline points, and then, the blank filter spectrum was used to correct each sample spectrum by simple blank subtraction [11]. The macro-output is the integrated peak area for each sample in two regions: 767 to 816 cm^−1^ corresponds to the characteristic peak area for (alpha) quartz, and 900 to 930 cm^−1^ corresponds to the primary peak area for kaolinite. (Notably, kaolinite also has a secondary peak that interferes with the quartz peak area. However, the secondary peak occurs in near constant proportion with the primary peak, and *FAST* has been programmed to correct for this.)

The integrated peak areas for quartz and kaolinite were output as a CSV file, which was then imported to the *FAST* software. As explained earlier, *FAST* does not include a model for the specific sampling conditions used in this study (i.e., cyclone-cassette assembly with built-in fitting as shown in Fig. 1f, with airflow at 2.0 L/min). However, it does include a model for the same hardware at 1.7 L/min—called “10-mm nylon Dorr-Oliver cyclone (with built-in fitting)” as shown in Fig. 1c. This model was selected to analyze the mine filter sample data, and a subset of the samples were also analyzed by a standard method for reference (see below). The *FAST*-output quartz mass (called “silica mass” in the software) was recorded for each filter sample and used to compute TWA quartz concentration using Eq. (6):

6 $${C}_{Q\_FAST} \mathrm{(}\frac{\mu {\mathrm{g}}}{{\mathrm{m}}^{3}}\mathrm{)}=\frac{1.38 \times {M}_{Q\_FAST} \mathrm{(}\mu \mathrm{g)}}{2.0 \left(\frac{\mathrm{L}}{{\mathrm{min}}}\right) \times t \mathrm{(min)}}\times \frac{1000 {\mathrm{L}}}{{\mathrm{m}}^{3}}$$

where ${M}_{Q\_FAST}$ is the quartz mass (μg) output by *FAST*; 1.38 is the constant MRE-equivalency factor; *t* is the sampling duration (min) for the CMDPSU; and 2.0 L/min is the sampling flow rate for the CMDPSU. Notably, for quartz measurements determined with the portable FTIR instrument on 37-mm PVC filters, *FAST* considers the limit of detection (LOD) and limit of quantification (LOQ) to be 5 μg and 16 μg, respectively [11] (NIOSH, 2022). These limits are based on analysis of blank filters [12], and the software notifies the user if the measured value for a sample is below the LOD or LOQ.

The respirable dust samples generated in the lab from Mine 28 dust source materials were also analyzed by the portable FTIR instrument, using the same procedure described above to measure the integrated areas for the quartz and primary kaolinite peaks (i.e., following baseline and blank filter corrections). However, to determine quartz mass on each filter, an internally developed algorithm was used rather than *FAST*. This is because the lab-generated filter samples were collected (a) using 2-piece cassettes from (b) a high-concentration enclosure. The combination of these two factors leads to a deposition pattern that is substantially more center-heavy than the pattern which occurs with 3-piece (and 4-piece) cassettes in a typical mine environment, and therefore none of the *FAST* models are expected to accurately predict quartz mass. The internal algorithm to determine quartz mass on these filters essentially had two steps: First, the measured quartz peak area was corrected for kaolinite interference per [21]. Second, the corrected quartz peak area was translated to quartz mass using the calibration curve given by [22]. Equation (7) combines these two steps to compute quartz mass on the 2-piece lab filters:

7 $${M}_{Q\_2PC}\text{ (}\mu \mathrm{g)}=\frac{{(P}_{Q}- \frac{{P}_{K}}{3.8})}{0.00695 ({\mu {\mathrm{g}}}^{-1})}$$

where *P*_*Q*_ and *P*_*K*_ are the integrated areas (arbitrary units) of the quartz and primary kaolinite peaks, respectively; 3.8 is a constant ratio between the primary and secondary kaolinite peak areas [21]; and 0.00695 (arbitrary units/μg) is the slope of a calibration curve for (kaolinite-corrected) integrated quartz peak area versus measured quartz mass, determined in the laboratory for filter samples collected in 2-piece cassettes at high dust concentration [22].

## Reference Quartz Analysis

**Fig. 4:**
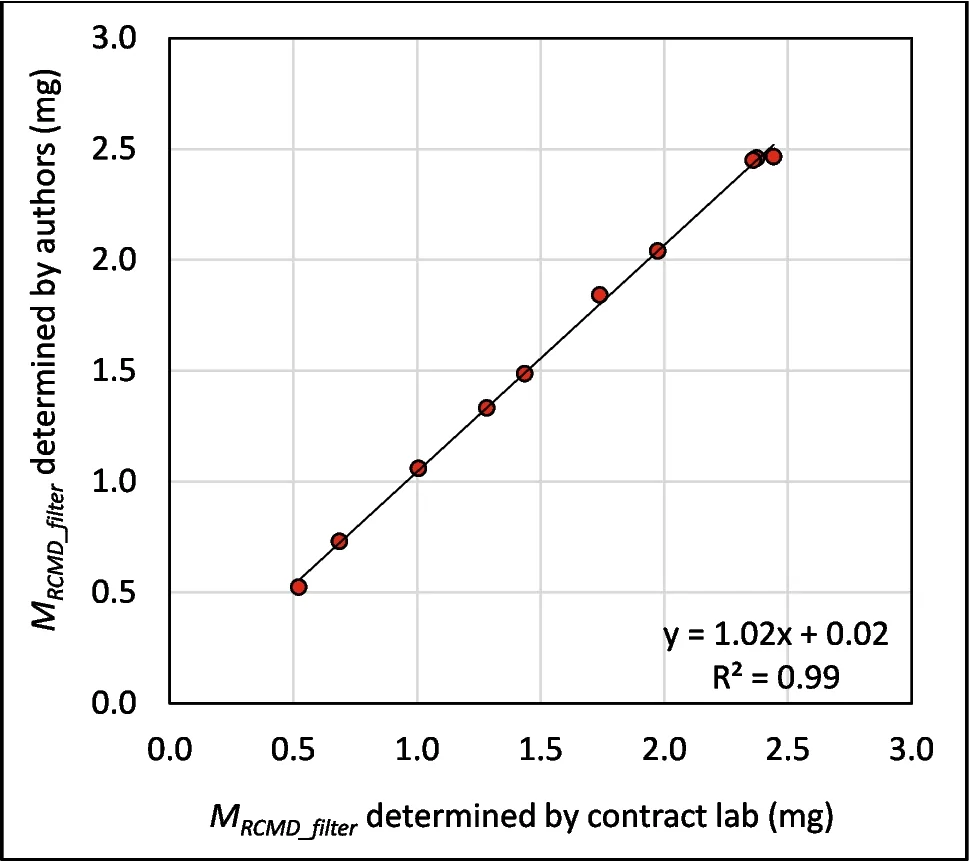
RCMD mass determined by the authors versus the contract lab for a subset of 10 filter samples selected for method 7603 analysis

Ten of the Mine 28 filter samples were selected for analysis by standard NIOSH Method 7603 [7]; this analysis was conducted by a contract lab (RJ Lee Group, Pittsburgh, PA). These samples were selected to represent a range of sampling events and sample masses. Prior to performing the Method 7603 analysis, the contracted lab weighed each filter (i.e., to re-measure *F*_*2*_ and determine ${M}_{RCMD\_filter}$ using the *F*_*1*_ values provided by the authors*.*) Fig. 4 shows the correlation between sample mass determined by the authors versus the contract lab, which confirms that sample loss and/or contamination was unlikely during transport.

The contract lab reported the 7603-derived quartz mass (μg) for each of the 10 filters, and then the (MRE-equivalent) TWA quartz concentration was computed using Eq. (8) (analogous to Eq. (6) for the FAST-derived quartz mass values).

8 $${C}_{Q\_7603} \mathrm{(}\frac{\mu {\mathrm{g}}}{{\mathrm{m}}^{3}}\mathrm{)}=\frac{1.38 \times {M}_{Q\_7603} \mathrm{(}\mu \mathrm{g)}}{2.0 \left(\frac{\mathrm{L}}{{\mathrm{min}}}\right) \times t \mathrm{(min)}}\times \frac{1000 {\mathrm{L}}}{{\mathrm{m}}^{3}}$$

### Quartz Percentage

For the Mine 28 filter samples, quartz percentage was determined as the ratio of quartz concentration to RCMD concentration per Eq. (9):

9 $${Q}_{RCMD}\left(\%\right)=100\% \times \frac{{C}_{Q}(\frac{\mu {\mathrm{g}}}{{\mathrm{m}}^{3}})}{{C}_{RCMD}(\frac{\mathrm{mg}}{{\mathrm{m}}^{3}})} \times \frac{\mathrm{mg}}{\text{1000 }\mu {\mathrm{g}}}$$

where ${C}_{Q}$ (μg/m^3^) is the TWA quartz concentration that is derived either from the DOF FTIR method or 7603 derived, and ${C}_{RCMD}$ (mg/m^3^) is the TWA RCMD concentration that is either derived from gravimetric analysis of the filter sample or estimated from the paired CPDM instrument(s).

For the respirable dust samples generated in the lab using dust source materials from Mine 28, quartz percentage was determined using Eq. (10):

10 $${Q}_{RD}=100\%\times \frac{{M}_{Q\_2PC}\mathrm{(}\mu \mathrm{g)}}{{M}_{RD} ({\mathrm{mg}})} \times \frac{\mathrm{mg}}{\text{1000 }\mu {\mathrm{g}}}$$

## Results and Discussion

A summary of the gravimetric and DOF FTIR analysis on the Mine 28 filter samples (i.e., collected with the CMDPSUs) is given in Table S2, and a summary of the CPDM data is given in Table S3. For three sample sets (*n* = 9 filter samples)—all of which were collected in the section intake (Location 4 per Fig. 2)—the post-weight measurements on all filters indicated negligible sample mass (i.e., ${M}_{RCMD\_filter}$ < 10 μg). Thus, these filters were excluded from further analysis (i.e., yielding a total of 63 filter samples in 21 sets). Notably, the CPDM data from these sample sets also indicated negligible dust concentration in the sampling environment (see Table S3). Several other CPDM data logs showed error or warning messages (also in Table S3): HIGH FILTER LOAD (*n* = 1), which occurs when the TEOM filter is nearing its maximum capacity; MASS OFFSET (*n* = 6), which occurs in response to rapid weight gain/loss from the TEOM filter; and POWER LOW (*n* = 2), which occurs when the CPDM battery is low [23]. In these instances, data were still used to estimate ${C}_{RCMD\_CPDM}$ and the results are clearly marked in related figures and tables.

**Fig. 5:**
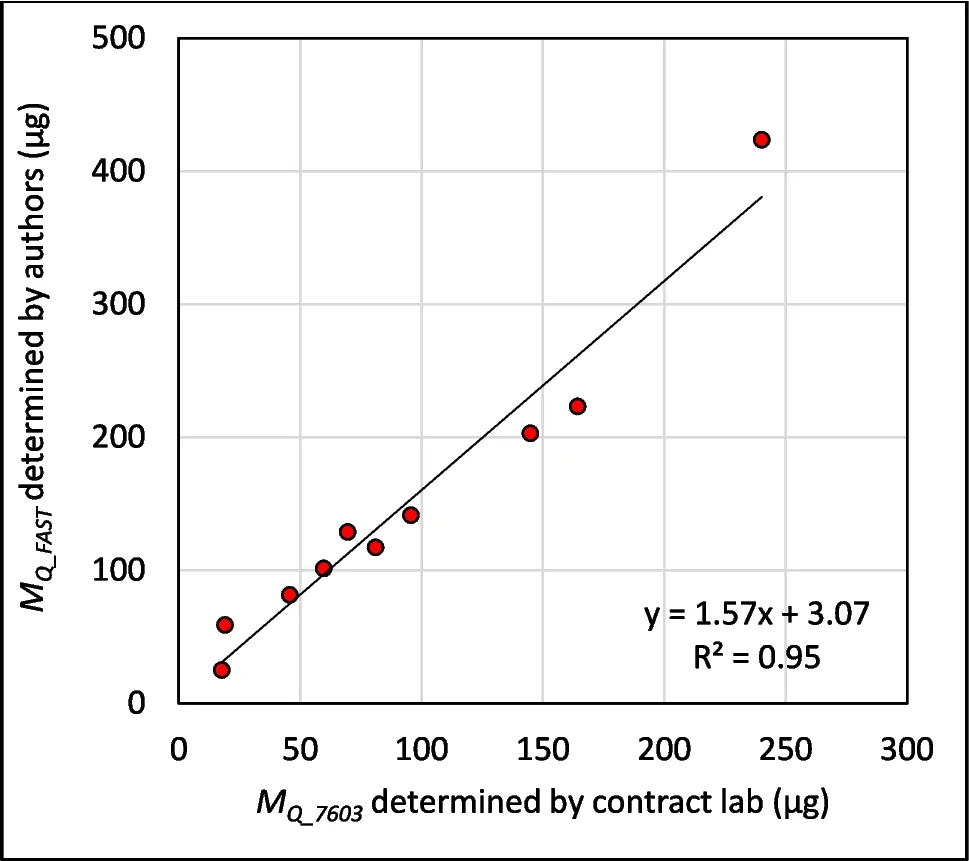
FAST-derived quartz mass versus method 7603 quartz mass for a subset of 10 filter samples

As mentioned, the filter samples in Mine 28 were collected with the cyclone-cassette assembly shown in Fig. 1f at 2.0 L/min (i.e., standard for RCMD sampling with the CMDPSU). However, since no *FAST* model exists for these conditions, the model shown in Fig. 1c was used to generate quartz mass values. To interpret these results, a correction was established by comparing the FAST-output values to the reference measurements (i.e., Method 7603 analysis) for the subset of 10 samples analyzed by both methods (Table S4). Figure 5 shows the correlation plot. There is a clear linear relationship, and the line of best fit shows little scatter (coefficient of determination, *R*^2^, is 0.95) and a minimal y-intercept (about 3 μg). These results provide further evidence that the sample handling and transport did not appreciably affect quartz measurements. However, the trendline slope in Fig. 5 is approximately 1.6, meaning that the *FAST* model used here tends to overpredict quartz mass by a factor of 1.6. This is attributed to the fact that the *FAST* model assumes a sampling flow rate of 1.7 L/min. The higher flow rate used in this study should yield a deposition pattern that is somewhat heavier than the filter center, which is the scan location for the DOF FTIR analysis. The *FAST* model extrapolates data from this location to the entire filter area based on expected deposition pattern; so, if more of the total dust is within the scan area than expected, the model prediction will be too high. Nevertheless, Fig. 5 demonstrates that the *FAST*-output quartz mass can be reasonably corrected by a factor of 1.6, over the mass range represented by filter samples collected for this field study. Thus, Eq. (6) was modified to Eq. (11) to incorporate the correction factor.

11 $${C{\prime}}_{{Q}_{FAST}} \mathrm{(}\frac{\mu {\mathrm{g}}}{{\mathrm{m}}^{3}}\mathrm{)}=\frac{1.38 \times {M}_{Q\_FAST} \mathrm{(}\mu \mathrm{g)}}{1.6 \times 2.0 \left(\frac{\mathrm{L}}{{\mathrm{min}}}\right) \times t \mathrm{(min)}}\times \frac{1000 {\mathrm{L}}}{{\mathrm{m}}^{3}}$$

### Trends in Quartz Concentration and Percentage in Study Mine

**Fig. 6:**
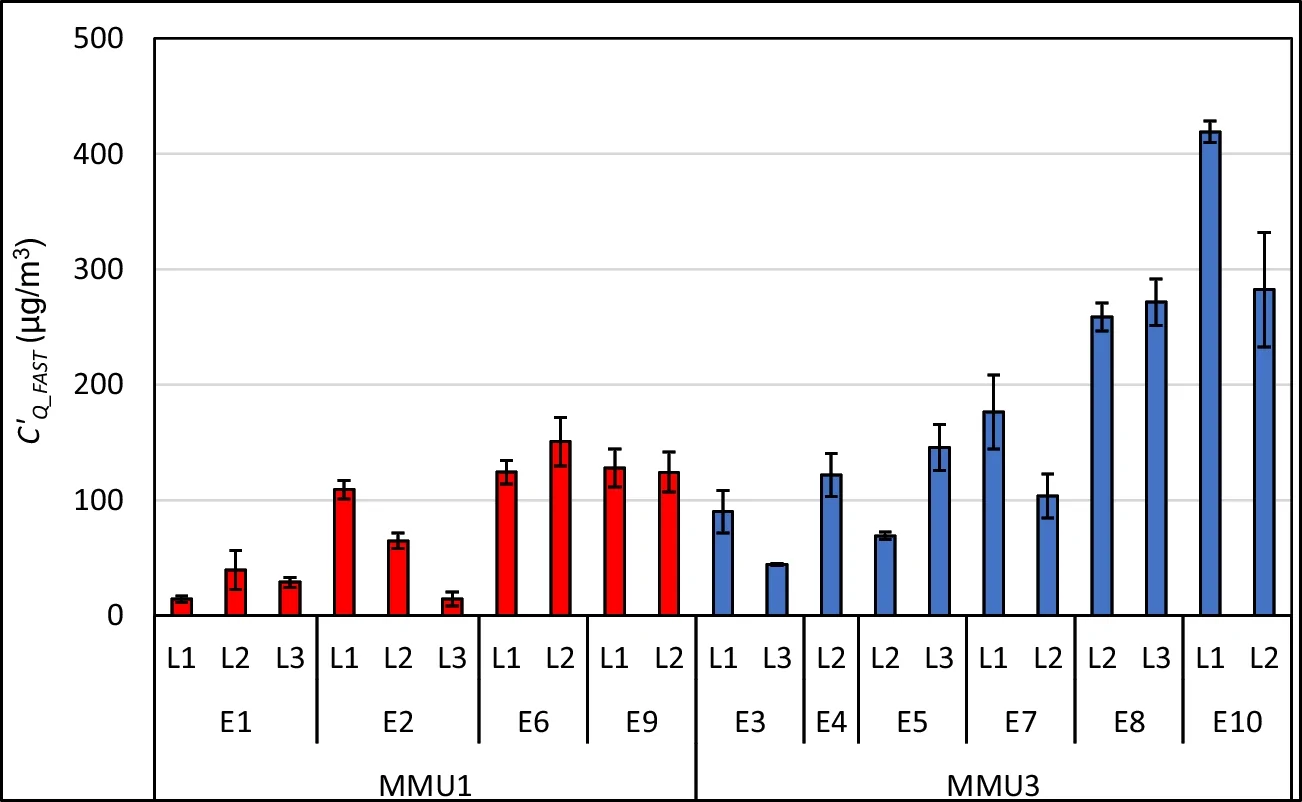
Corrected *FAST*-derived quartz concentration for each sample set. Results are presented as the mean and standard deviation (error bars) for each set of triplicate filters

(Fig. 6 shows the quartz concentration C’_QFAST_ in each sampling event and location (*n* = 21 sample sets). As mentioned, the samples collected in the intake location (L4) did not have enough mass for analysis in either MMU. Regarding the other samples, it should be reiterated that these were collected in fixed locations—including in areas that mine personnel are generally not expected to be working in regularly or for long durations (i.e., just downwind of the continuous miner or in the section return). Thus, results presented here should not be construed as representative of typical exposures. Rather, this sort of sampling scheme could be used for collection of routine non-regulatory samples, for example, to track trends in quartz concentration with mining or geological conditions.

(Fig. 6 shows no discernable trends among samples collected in L1, L2, and L3. For instance, in events 2, 3, 7, and 10, the quartz concentration was clearly higher downwind the continuous miner (L1) than in the section return (L2) and/or just downwind the bolter (L3), but the opposite is true for events 1 and 6. Similarly, there was not a consistent trend between L2 and L3. In general, the quartz concentration increased with event number in both mine sections sampled—although there is no specific explanation for this in terms of the activities or conditions in the mine (e.g., production, ventilation, strata conditions).

Nevertheless, Fig. 6 shows that the quartz concentration was higher on average in MMU3 than in MMU1 (i.e., 180.3 μg/m^3^ versus 79.8 μg/m^3^, respectively); Student’s *t*-test comparing group means (α= 0.05, assuming unequal variances) indicated the difference was significant (*P* = 0.02). This finding was unexpected based on the observation that MMU3 was mining relatively little roof rock along with the coal seam, whereas substantial rock was being taken in MMU1 (see Table S1). For context, the average height of roof rock being mined represented about 27% of the total mining height during the four sampling events in MMU1, but only about 7% during the six events in MMU3. However, analysis of the dust source materials helps explain the results: the respirable dust generated from the coal being mined in MMU3 contained about 7.4% quartz, whereas this value was only about 0.9% for the coal in MMU1 (Table S5). For the respirable dust samples representing the roof rock strata, results indicated quartz content was about 19.3% in MMU1 and 15.1% in MMU3. (The rock dusting product, which was used in both sections, was found to have a quartz content of about 2.5%. However, based on field observations, the rock dusting product was not expected to be a substantial source of the RCMD in locations sampled for this study.)

**Fig. 7:**
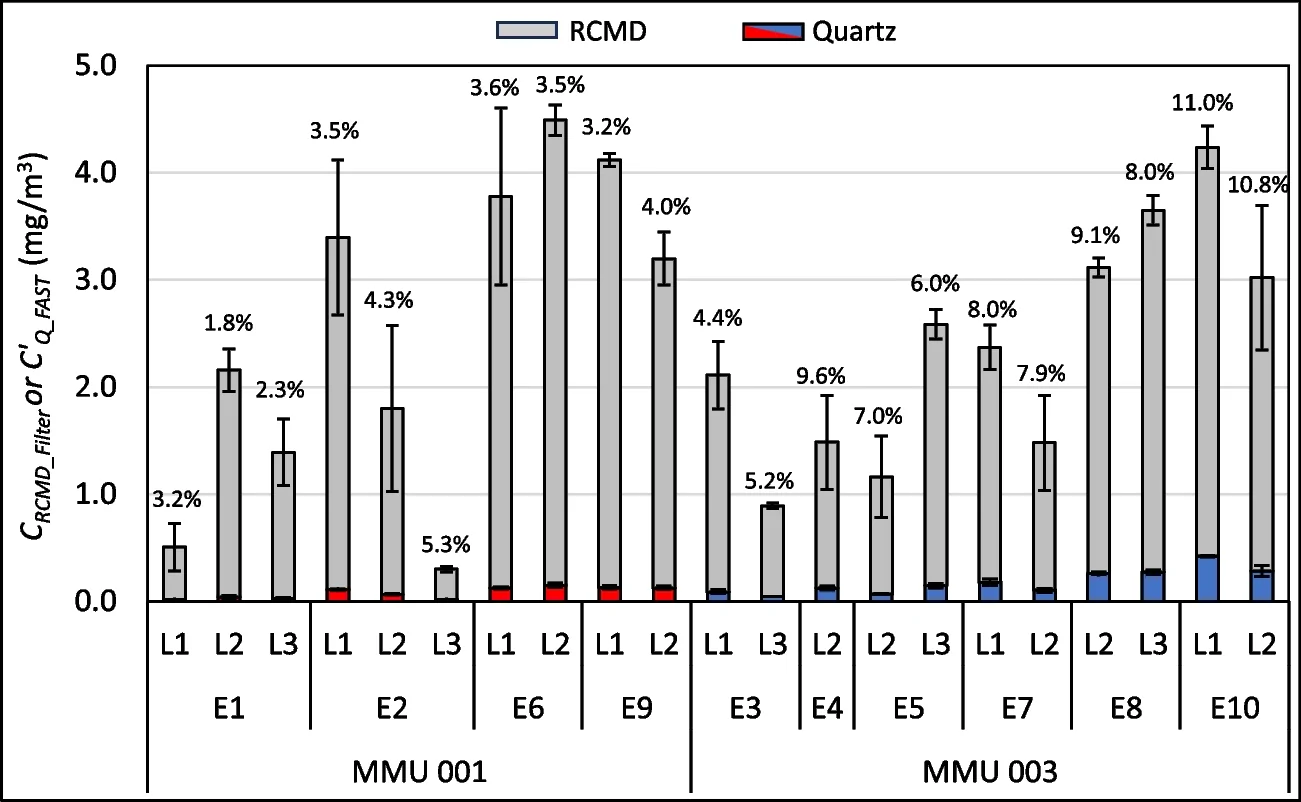
RCMD concentration (derived from gravimetric filter analysis) and corrected *FAST*-derived quartz concentration (expressed as mg/m^3^) for each sample set. Concentration results are presented as the mean and standard deviation (error bars) for each set of triplicate filters. The mean quartz percentage for each set is also annotated

To put the quartz concentration data into context, Fig. 7 plots this data along with the total RCMD concentration (${C}_{RCMD\_filter}$) and quartz percentage (${Q}_{RCMD}$), determined using only the filter samples (i.e., not considering the CPDM data). While samples from MMU1 generally indicated lower quartz concentrations than in MMU3, they indicated slightly higher RCMD concentrations (i.e., average of 2.7 mg/m^3^ versus 2.2 mg/m^3^, respectively), but the difference was not found to be statistically significant (*P* = 0.65). Prior studies have found that, with continuous mining, cutting rock—which is generally much harder than the target coal seam—can produce substantially more respirable dust than cutting coal [24]. However, the personnel in Mine 28 noted that the coal seam in both sections was particularly hard, which could limit relative differences in the amount of dust being generated from cutting the roof rock versus the coal.

Still, Fig. 7 shows that the percentage of quartz in the RCMD (${Q}_{RCMD}$) was higher in MMU3 than in MMU1 (i.e., 7.9% versus 3.9%, respectively) and this difference was found to be statistically significant (*P* < 0.001). Again, this is attributed to the relatively high quartz content of the coal being mined in MMU3 during the field study. A very simple source apportionment exercise is illustrated in Table 2, considering only the average heights of coal and roof rock being mined in each section and the quartz content of those strata (i.e., based on the analysis of respirable dust generated from the grab samples of source materials). Assuming that during the field study (a) the continuous miner operation was the predominant source of RCMD, (b) the coal and rock strata produced similar amounts of RCMD, and (c) the respirable quartz content of these strata was represented by the samples generated in the lab, the predicted ${Q}_{RCMD}$ would be about 5.8% in MMU1 and 7.9% in MMU3. Despite the simplicity of this exercise, the results are consistent with observations based on the RCMD filter sample analysis. Indeed, this sort of sampling approach (i.e., collecting dust source materials along with RCMD samples) could be useful for mines aiming to identify and understand variability in RCS sources.

**Table 2:** Simple quartz source apportionment exercise to predict quartz content in RCMD based on quartz content in primary geologic strata and relative mining heights in those strata

Section	Stratum height (ft)		Quartz content (%)		Predicted Q_RCMD_ (%)
	**Coal (** ${{\boldsymbol{H}}}_{{\boldsymbol{c}}{\boldsymbol{o}}{\boldsymbol{a}}{\boldsymbol{l}}}$**)**	**Rock (** ${{\boldsymbol{H}}}_{{\boldsymbol{r}}{\boldsymbol{o}}{\boldsymbol{c}}{\boldsymbol{k}}}$**)**	**Coal (** ${{\boldsymbol{Q}}}_{{\boldsymbol{R}}{\boldsymbol{D}}\_{\boldsymbol{c}}{\boldsymbol{o}}{\boldsymbol{a}}{\boldsymbol{l}}}$**)**	**Rock (** ${{\boldsymbol{Q}}}_{{\boldsymbol{R}}{\boldsymbol{D}}\_{\boldsymbol{r}}{\boldsymbol{o}}{\boldsymbol{c}}{\boldsymbol{k}}}$**)**	$=\frac{{({\boldsymbol{H}}}_{{\boldsymbol{c}}{\boldsymbol{o}}{\boldsymbol{a}}{\boldsymbol{l}}}\times {{\boldsymbol{Q}}}_{{\boldsymbol{R}}{\boldsymbol{D}}\_{\boldsymbol{c}}{\boldsymbol{o}}{\boldsymbol{a}}{\boldsymbol{l}}})+{({\boldsymbol{H}}}_{{\boldsymbol{r}}{\boldsymbol{o}}{\boldsymbol{c}}{\boldsymbol{k}}}\times {{\boldsymbol{Q}}}_{{\boldsymbol{R}}{\boldsymbol{D}}\_{\boldsymbol{r}}{\boldsymbol{o}}{\boldsymbol{c}}{\boldsymbol{k}}})}{{({\boldsymbol{H}}}_{{\boldsymbol{c}}{\boldsymbol{o}}{\boldsymbol{a}}{\boldsymbol{l}}}+{{\boldsymbol{H}}}_{{\boldsymbol{r}}{\boldsymbol{o}}{\boldsymbol{c}}{\boldsymbol{k}}})}$
MMU1	5	1.8	0.9	19.3	5.8
MMU3	7.8	0.5	7.4	15.1	7.9

### Estimating Quartz Percentage with CPDM Data

**Fig. 8:**
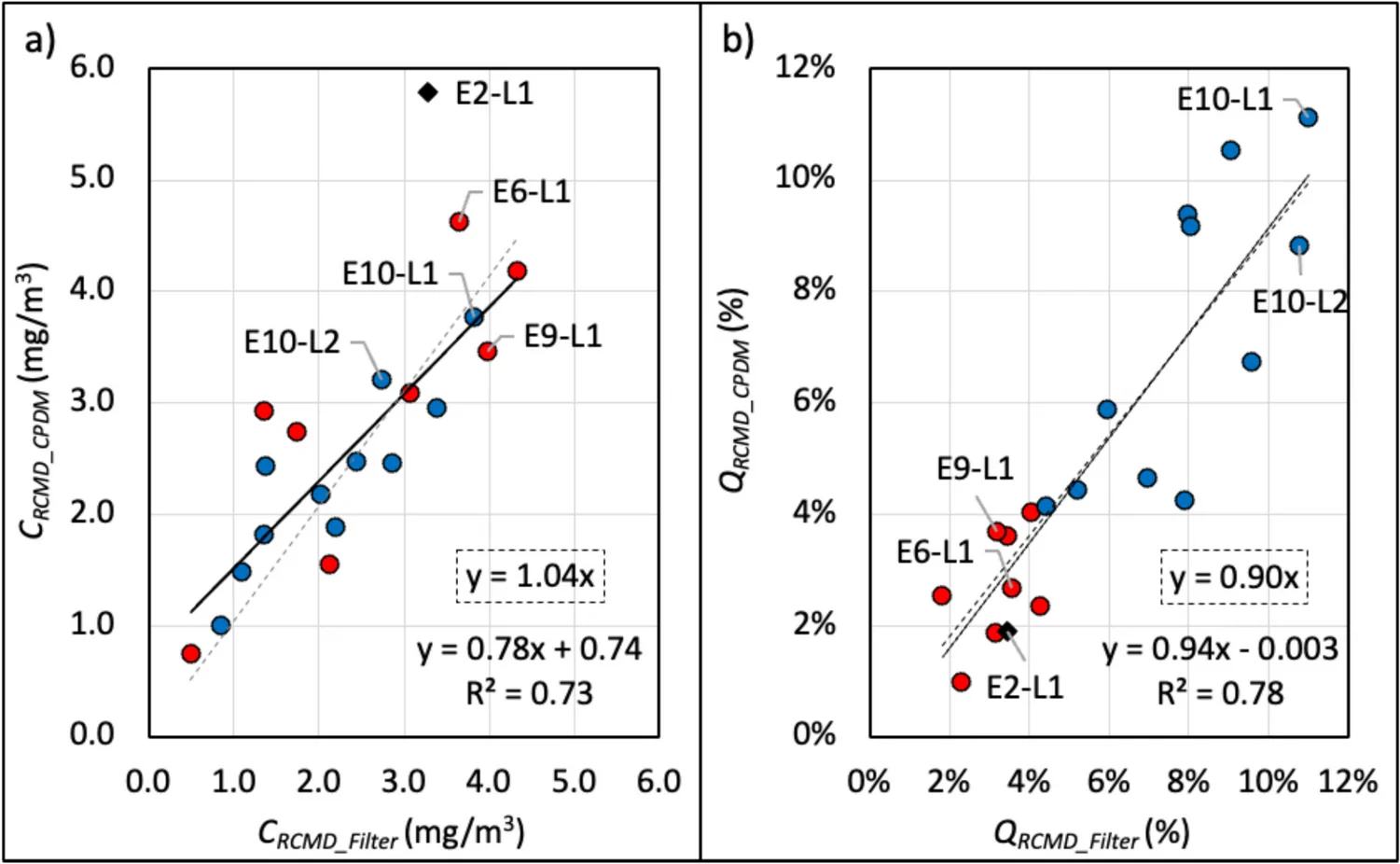
Comparison **a** RCMD concentration measured by CPDM versus gravimetric analysis of filter samples for each sample set and **b** the resultant quartz percentage values. The five labeled datapoints on each plot represent the sample sets where data logs from one or both of the CPDMs indicated warnings or errors; the E2-L1 datapoint was determined to be an outlier and excluded from trendline fitting

In Fig. 7, the quartz percentage data is based solely on analysis of the CMDPSU filter samples, including post-weighing the filters to determine RCMD mass concentration (i.e., ${C}_{RCMD\_filter}$ per Eq. (2)). However, this approach is impractical in the field since a microbalance is unlikely to be available. Figure 8 evaluates the option to instead use a paired CPDM instrument as a means to estimate the RCMD concentration for a filter sample (i.e., ${C}_{RCMD\_CPDM}$ per Eq. (4)). Figure 8 a compares the CPDM- versus filter sample-derived RCMD concentration for each sample set (i.e., one or two CPDMs and three CMDPSUs per set; *n* = 20 sets per Table S3). (It is reiterated that, for both measures, the values represent MRE-equivalent TWA concentration.) Excluding just one datapoint which appears to be an outlier (i.e., value is greater than the third quartile for all points by more than 1.5 × the inter-quartile range), there is strong correlation between the two measures (*R*^2^ = 0.73). This is expected since the CPDM was developed using standard CMDPSU sampling as the reference [20, 25]. Notably, the linear trendline of best fit for this dataset has a slope of 0.78 and a y-intercept of 0.74 mg/m^3^, which implies the CPDM would measure non-trivial RCMD concentration even when a filter sample indicates negligible mass. Notably, if the trendline were constrained to a y-intercept value of 0 (as suggested by [25]), the slope would be 1.04 (see dashed trendline in Fig. 8a).

In Fig. 8b, the quartz percentage for each sample set (i.e., ${Q}_{RCMD}$ per Eq. (9)) is compared for values derived using the CPDM versus filter sample analysis. Again, the correlation is strong (*R*^2^ = 0.78) and the line of best fit has a slope of 0.94 and minimal y-intercept.

Taken together, the trends shown in Fig. 8 suggest that, on average, the CPDM instruments used in Mine 28 slightly overestimated the RCMD concentration, which resulted in a slight underestimation of the quartz percentage (i.e., due to the inverse relationship between ${Q}_{RCMD}$ and ${C}_{RCMD}$). Nevertheless, assuming that CPDMs are maintained and calibrated (as they were in Mine 28), these results suggest that the paired CPDM approach could work well to enable quartz percentage estimates in the field for non-regulatory monitoring purposes.

### Feedback from Mine Personnel

During the sampling campaign for the field study, a demonstration of the DOF FTIR was conducted with the mine engineers and the health and safety team at Mine 28, including sample handling (i.e., transferring the sample filter from the 3-piece cassette to the analysis cassette); FTIR analysis of the sample filter (and blank); baseline and blank correction of the sample spectrum (i.e., in the OPUS software that goes with the Bruker FTIR instrument used in this study); download of the sample data from the FTIR software and upload to *FAST* software; and use of *FAST* to generate quartz results. With the exception of the sample handling step needed for this study (i.e., due to the choice to demonstrate 3-piece rather than the special 4-piece sampling cassettes), these steps are all explained in detail in NIOSH’s guide for the DOF FTIR method [11].

Immediate feedback from the mine personnel was generally positive, with specific points being the efficiency and convenience of the DOF FTIR method. A main point of concern was the need to use CMDPSUs for sampling. Like many mines, Mine 28 has generally not collected filter samples since the CPDM was mandated for operator-conducted RCMD sampling in 2016, and, in fact, mine personnel were unaware of any CMDPSUs that may still be on site. The mine personnel expressed interest in capabilities for field analysis of CPDM samples, though no such method presently exists and a DOF FTIR approach appears improbable with the current CPDM filter assembly, which includes borosilicate glass fiber material that directly interferes with the quartz spectral peak area [26, 27].

In follow-up discussions with the mine partner (i.e., Mine 28 operator), they expressed interest in using the DOF FTIR method for non-regulatory RCS monitoring—and there is a sense of urgency given the reduced PEL set by the “new silica rule” [5]. For example, this mine partner has an interest to collect baseline quartz concentration data under different mining/geologic conditions and to test new or modified dust control conditions (i.e., beyond what is required by their current ventilation plans). The partner has made clear that their interest would be to conduct such studies using area samples in order to isolate test variables and avoid personal sampling factors that could confound observations (e.g., specific positioning or actions of individual workers).

### Study Implications and Limitations

This field study demonstrates use of DOF FTIR for RCS monitoring in a coal mine setting, using a modified approach with respect what has been previously tested by NIOSH or others. Notably, the study objectives were outlined with input from the Mine 28 operator—and all field samples were collected—prior to publication of the “new silica rule.” Accordingly, the CMDPSU sampler and 2.0 L/min flow rate were chosen to match the conventional RCS monitoring conditions, and the specific combination of cassette and cyclone-cassette assembly was chosen to enable accurate DOF FTIR results with inexpensive off-the-shelf components (i.e., 3-piece cassettes, expandable assembly with built-in fitting from cyclone to cassette). These conditions provided the impetus to use the “closest” *FAST* model and establish a correction factor for the results. Moreover, the aim to estimate RCS percentage (in addition to mass concentration) in the field led to use of the paired CPDMs.

Although the “new silica rule” may reframe some of the work presented here (e.g., in terms of filter samplers and sampling conditions that will be used for compliance sampling), several lessons from this study can be carried forward to support non-regulatory monitoring with DOF FTIR. Importantly, personnel responsible for sample collection and analysis must be knowledgeable of the underlying principles and assumptions. As shown here, the specific sampler components (e.g., cassette, cyclone and the assembly of the two) and conditions (e.g., flow rate) are critical to the dust deposition pattern on the filter, which must be accurately modeled to translate the raw FTIR spectral results to quartz mass. Thus, while *FAST* provides a user-friendly interface for analysis of the raw FTIR results, the user must determine when to use one of the existing *FAST* models outright, or when a correction factor might be needed. When needed, the current study illustrates a basic strategy for calibration of *FAST* results against reference measurements on a subset of sample filters. This strategy would be advisable as a preliminary or periodic check on DOF FTIR measurements under any sampling conditions, and could provide estimates of method uncertainty (e.g., quantified by standard error of prediction). The specific correction factor established here might be useful for future efforts to compare RCS concentrations derived from filters collected using “old” versus “new” sampling conditions (i.e., CMDPSU at 2.0 L/min versus CMDPSU at 1.7 L/min or any other ISO 7708-compliant sampler), or in instances where the paired CPDM approach is used to allow estimation of quartz percentage (i.e., since the CPDM was designed to match RCMD concentration measured by a CMDPSU at 2.0 L/min). Of course, some knowledge of FTIR principles is also needed to ensure reliable FTIR data generation, detection and troubleshooting of possible errors, and communication of results.

Further, users must consider the availability of suitable space for housing the FTIR instrument and conducting the analysis; in general, this space should be relatively free of dust and climate controlled (i.e., to limit humidity). Care must also be taken during sample handling to minimize the potential for sample loss or contamination. That said, the current study demonstrates that this is possible, even using the 3-piece styrene cassettes which require the filter sample to be transferred to a separate cassette for analysis. Notably, the researcher performing the DOF FTIR sample analysis here had no prior experience with collecting or handling such filter samples.

In terms of study limitations, several should be noted. First, this work represents a limited sampling campaign in single mine. The sampling locations were chosen to allow analysis of major dust sources and their silica component across the main air course, and especially in locations L1, L2, and L3, samplers were positioned to maximize dust collection. Thus, while estimated quartz *percentages* might be relevant to personal exposures in the vicinity of each sampling location during the collection periods, the quartz *concentrations* cannot be interpreted with respect personal exposures. It is also reiterated the quartz source apportionment exercise (Table 2) is meant to offer only general insights, since it considers just a single bulk sample of each primary dust source material collected from each mine section. Regarding use of the paired CPDM filter sampler approach, it must be acknowledged that spatial variability in the sampling environment can affect results. This might explain some of the scatter evident in Fig. 8, and indeed there was even variability between some sets of triplicate filters (see error bars in Figs. 6 and 7). Effects of spatial variability could be reduced in future field studies by using “can” samplers [28].

## Conclusions

There is a clear need for more frequent RCS monitoring in coal mines, especially using methods which can yield timely data. NIOSH has pioneered DOF quartz analysis by FTIR for this purpose, and developed the freely available *FAST* software to simplify data processing. However, adoption by mines may depend on a range of factors including costs, ease of use, and the usefulness and reliability of data. This field study sought to demonstrate the DOF FTIR method with *FAST* under specific conditions that mines might prefer for routine non-regulatory monitoring. These include use of low-cost, widely available 3-piece sampling cassettes in combination with an expandable cassette-cyclone holder assembly, which can be used as-designed; and use of a CPDM instrument paired with the CMDPSU filter sampler (at 2.0 L/min) to enable estimation of quartz percentage (in addition to quartz mass).

The field study results show that sampling with 3-piece cassettes and the expandable cyclone-cassette holder is indeed a viable alternative to the specific cassette and cyclone-cassette holder combinations that have been specifically included in *FAST* for coal mine monitoring. In this case, the *FAST-*output results must be corrected to account for a difference in dust deposition pattern yielded by the alternative sampling conditions (versus those modeled in *FAST*). Here, it was demonstrated that the correction factor can be easily determined by comparing the *FAST-*output results to those of the standard NIOSH Method 7603 (analogous to MSHA Method P7) for a limited number of filter samples. And, at least for the range of quartz masses encountered in this field study, the correction factor was found to be constant.

Further, the paired CPDM approach to enable field estimation of quartz percentage on filter samples was shown to be generally valid. In essence, this approach uses the DOF FTIR method to determine quartz concentration from the filter sample, and the paired CPDM is used to estimate respirable dust concentration. Quartz percentage data may be particularly useful in cases where mines are seeking to identify RCS sources or track trends with specific geologic or mining conditions.

## Supplementary Information

Below is the link to the electronic supplementary material.

Supplementary file1 (DOCX 92.4 KB)

## Data Availability

All data associated with this paper is included within this paper or the associated supplemental information.

## Footnotes

1 From Zefon International (Ocala, FL), which manufacturers the 4-piece cassettes: for pre-loaded cassettes (37-mm PVC filter and support), styrene 3-piece cassettes were just under $2/each ($94.50 per box of 50), whereas conductive 4-piece cassettes were just under $14/each ($138 per box of 10).
