# Supplementary material for: Direct-on-Filter FTIR Analysis of Respirable Crystalline Silica: A Field Study to Demonstrate Utility for Routine Non-regulatory Monitoring in Coal Mines
Source: Min Metall Explor. 2024 Dec 5;41(6):2817–31. doi: 10.1007/s42461-024-01154-4 (PMC13582105; doi:10.1007/s42461-024-01154-4)
Supplement: Supplementary file 1 [file 42461_2024_1154_MOESM1_ESM.docx]

# Supplemental Information

Table S1: Summary of sampling events in Mine 28. *Anemometer unavailable.

| **Event and MMU** | | **Location** | | **Date** | **Time** | | **Duration (min)** | **CPDM Number** | **Average Mining height (ft)** | | **Airway dimensions (ft)** | | **Velocity (ft/min)** | **Airflow (ft^3^/min)** |
| --- | --- | --- | --- | --- | --- | --- | --- | --- | --- | --- | --- | --- | --- | --- |
|  |  |  |  |  | **Start** | **End** |  |  | **Coal** | **Rock** | **Width** | **Height** |  |  |
| E1 | 1 | L1 | Entry 0, XC-64 Left | 7/10/2023 | 16:47 | 21:40 | 293 | CPDM 12 | 4.58 | 2.23 | 18.42 | 6.17 | * | - |
| E1 | 1 | L2 | Entry 4, XC-63 Inby | 7/10/2023 | 17:00 | 21:41 | 281 | CPDM 14 |  |  | 16.50 | 7.67 | 192.0 | 24,288.0 |
| E1 | 1 | L3 | Entry 00, XC 64-65 Left | 7/10/2023 | 16:54 | 21:38 | 284 | CPDM 13 |  |  | 19.42 | 7.50 | * | - |
| E1 | 1 | L4 | Entry 2, XC-65 Left | 7/10/2023 | 16:42 | 21:35 | 293 | CPDM 11 |  |  | 19.83 | 6.75 | * | - |
| E2 | 1 | L1 | Entry 0000, XC-64 Inby | 7/11/2023 | 16:13 | 21:25 | 312 | CPDM 12 | 4.79 | 2.00 | 18.25 | 7.42 | 65.0 | 8,798.0 |
| E2 | 1 | L2 | Entry 0, XC-64 Outby | 7/11/2023 | 16:20 | 21:27 | 307 | CPDM 11 |  |  | 18.50 | 6.58 | 190.0 | 23,140.4 |
| E2 | 1 | L3 | Entry 1, XC-64 Outby | 7/11/2023 | 16:26 | 21:28 | 302 | NO CPDM |  |  | 17.83 | 6.83 | * | - |
| E2 | 1 | L4 | Entry 1, XC-64-65 Left | 7/11/2023 | 16:01 | 21:22 | 321 | CPDM 28 |  |  | 18.33 | 6.33 | 45.0 | 5,225.0 |
| E3 | 3 | L1 | Entry 0, XC 16 Outby | 7/12/2023 | 16:07 | 21:12 | 305 | CPDM 14, 27 | 7.58 | 1.04 | 17.83 | 9.42 | 183.0 | 30,459.3 |
| E3 | 3 | L3 | Entry 1, XC-15 Outby | 7/12/2023 | 16:00 | 21:10 | 310 | CPDM 11, 13 |  |  | 18.75 | 7.83 | 42.0 | 6,168.8 |
| E4 | 3 | L2 | Entry 1, XC-3 Outby | 7/13/2023 | 15:58 | 20:49 | 291 | CPDM 14, 27 | 7.67 | 0.33 | 17.75 | 8.33 | 180.0 | 26,625.0 |
| E4 | 3 | L4 | Entry 4, XC-2 Outby | 7/13/2023 | 16:07 | 20:51 | 284 | CPDM 11, 13 |  |  | 18.25 | 7.67 | 357.0 | 49,950.3 |
| E5 | 3 | L2 | Entry 0, XC-3 Outby | 7/14/2023 | 16:21 | 20:33 | 252 | CPDM 14, 27 | 8.13 | 0.38 | 18.58 | 8.92 | 207.0 | 34,300.2 |
| E5 | 3 | L3 | Entry 1, XC-5 Left | 7/14/2023 | 16:14 | 20:31 | 257 | CPDM 11, 13 |  |  | 17.42 | 8.08 | 154.0 | 21,680.9 |
| E6 | 1 | L1 | Entry 0000, XC-65 Inby | 7/17/2023 | 9:14 | 14:05 | 291 | CPDM 11, 13 | 4.79 | 1.88 | 19.83 | 6.92 | 69.0 | 9,465.5 |
| E6 | 1 | L2 | Entry 000-0000, XC-64 | 7/17/2023 | 9:20 | 14:07 | 287 | CPDM 14, 27 |  |  | 18.50 | 6.42 | 74.0 | 8,784.4 |
| E7 | 3 | L1 | Entry 0, XC-3 Inby | 7/18/2023 | 8:46 | 13:33 | 287 | CPDM 11, 13 | 8.33 | 0.04 | 17.42 | 8.42 | 163.0 | 23,894.2 |
| E7 | 3 | L2 | Entry 0, XC-5 Inby | 7/18/2023 | 8:39 | 13:35 | 296 | CPDM 14, 27 |  |  | 18.42 | 8.33 | 181.0 | 27,778.5 |
| E8 | 3 | L2 | Entry 0, XC-5 Inby | 7/19/2023 | 9:10 | 13:26 | 256 | CPDM 12, 19 | 7.75 | 0.42 | 18.67 | 8.08 | 189.0 | 28,518.0 |
| E8 | 3 | L3 | Entry 0-1, XC-7 | 7/19/2023 | 9:00 | 13:28 | 268 | CPDM 8, 10 |  |  | 18.25 | 8.25 | 201.0 | 30,263.1 |
| E9 | 1 | L1 | Entry 10, XC-67 Outby | 7/20/2023 | 9:32 | 13:47 | 255 | CPDM 8,10 | 6.00 | 0.50 | 17.50 | 6.08 | 220.0 | 23,420.8 |
| E9 | 1 | L2 | Entry 10, XC-66 Outby | 7/20/2023 | 9:23 | 13:46 | 263 | CPDM 12, 19 |  |  | 17.33 | 6.92 | 243.0 | 29,133.0 |
| E10 | 3 | L1 | Entry 0, XC-8 Inby | 7/21/2023 | 8:36 | 14:02 | 326 | CPDM 8,10 | 7.38 | 0.54 | 13.75 | 8.08 | 61.0 | 6,779.9 |
| E10 | 3 | L2 | Entry 0, XC-7 Outby | 7/21/2023 | 8:41 | 14:04 | 323 | CPDM 12, 19 |  |  | 19.17 | 7.75 | 164.0 | 24,360.8 |

Table S2: Mine 28 CMDPSU Filter Sample Data

|  |  |  |  | **RCMD** | | | | **Quartz** | | | | |  |  |
| --- | --- | --- | --- | --- | --- | --- | --- | --- | --- | --- | --- | --- | --- | --- |
| **Event** | **MMU** | **Location** | **Filter Number** | **M_RCMD_filter_ (mg)** | **Average M_RCMD_filter_ (mg)** | **C_RCMD_filter_ (mg/m^3^)** | **Average C_RCMD_filter_ (mg/m^3^)** | **M_Q_FAST_ (µg)** | **Average M_Q_FAST_ (µg)** | **C_Q FAST_ (µg/m^3^)** | **C'_Q FAST_ (µg/m^3^)** | **Average C'_Q FAST_ (µg/m^3^)** | **Q_RCMD_ (%)** | **Average Q_RCMD_ (%)** |
| E1 | 1 | L1 | S1 | 0.166 | 0.281 (± 0.127) | 0.290 | 0.492  (± 0.221) | 9.853 | 12.765 (± 2.498) | 17.347 | 10.842 | 14.047  (± 2.751) | 5.95% | 3.164%  (± 0.689%) |
|  |  |  | S2 | 0.218 |  | 0.386 |  | 12.489 |  | 21.983 | 13.740 |  | 5.73% |  |
|  |  |  | S3 | 0.458 |  | 0.800 |  | 15.953 |  | 28.097 | 17.561 |  | 3.48% |  |
|  |  | L2 | S1 | 1.173 | 1.190 (± 0.113) | 2.084 | 2.116  (± 0.198) | 24.592 | 35.544 (± 15.217) | 43.746 | 27.341 | 39.528  (± 16.925) | 2.10% | 1.818%  (± 0.608%) |
|  |  |  | S2 | 1.336 |  | 2.374 |  | 57.063 |  | 101.540 | 63.463 |  | 4.27% |  |
|  |  |  | S3 | 1.060 |  | 1.891 |  | 24.978 |  | 44.450 | 27.781 |  | 2.36% |  |
|  |  | L3 | S1 | 1.027 | 0.796 (± 0.182) | 1.753 | 1.362  (± 0.310) | 22.715 | 27.043 (± 4.029) | 38.750 | 24.219 | 28.839  (± 4.297) | 2.21% | 2.311%  (± 0.872%) |
|  |  |  | S2 | 0.781 |  | 1.339 |  | 25.999 |  | 44.367 | 27.729 |  | 3.33% |  |
|  |  |  | S3 | 0.581 |  | 0.994 |  | 32.416 |  | 55.310 | 34.569 |  | 5.58% |  |
|  |  | L4 | S1 | -0.035 | -0.018 (± 0.012) | - | - | - | - | - | - | - | - | - |
|  |  |  | S2 | -0.015 |  | - |  | - |  | - | - |  | - |  |
|  |  |  | S3 | -0.005 |  | - |  | - |  | - | - |  | - |  |
| E2 | 1 | L1 | S1 | 1.423 | 2.050 (± 0.451) | 2.277 | 3.284  (± 0.724) | 97.594 | 108.941 (± 8.278) | 156.395 | 97.747 | 109.118  (± 8.294) | 6.86% | 3.453%  (± 0.595%) |
|  |  |  | S2 | 2.262 |  | 3.629 |  | 112.125 |  | 179.690 | 112.306 |  | 4.96% |  |
|  |  |  | S3 | 2.466 |  | 3.947 |  | 117.107 |  | 187.680 | 117.300 |  | 4.75% |  |
|  |  | L2 | S1 | 1.739 | 1.068 (± 0.475) | 2.829 | 1.734  (± 0.774) | 72.933 | 63.760 (± 6.488) | 118.777 | 74.235 | 64.9  (± 6.603) | 4.19% | 4.258%  (± 1.155%) |
|  |  |  | S2 | 0.733 |  | 1.187 |  | 59.395 |  | 96.738 | 60.461 |  | 8.11% |  |
|  |  |  | S3 | 0.731 |  | 1.187 |  | 58.954 |  | 96.007 | 60.004 |  | 8.06% |  |
|  |  | L3 | S1 | 0.146 | 0.172 (± 0.018) | 0.248 | 0.285  (± 0.026) | 21.901 | 13.676 (± 5.868) | 36.266 | 22.667 | 14.15  (± 6.074) | 14.97% | 8.342%  (± 2.777%) |
|  |  |  | S2 | 0.184 |  | 0.304 |  | 10.516 |  | 17.402 | 10.876 |  | 5.73% |  |
|  |  |  | S3 | 0.186 |  | 0.304 |  | 8.611 |  | 14.255 | 8.910 |  | 4.62% |  |
|  |  | L4 | S1 | 0.005 | 0.014 (± 0.010) | - | - | - |  | - | - | - | - | - |
|  |  |  | S2 | 0.027 |  | - |  | - |  | - | - |  | - |  |
|  |  |  | S3 | 0.009 |  | - |  | - |  | - | - |  | - |  |
| E3 | 3 | L1 | S1 | 0.980 | 1.232 (± 0.190) | 1.601 | 2.019  (± 0.314) | 64.004 | 88.012 (± 17.968) | 104.921 | 65.576 | 90.174  (± 18.408) | 6.53% | 4.428%  (± 0.239%) |
|  |  |  | S2 | 1.437 |  | 2.360 |  | 107.226 |  | 175.771 | 109.857 |  | 7.46% |  |
|  |  |  | S3 | 1.281 |  | 2.098 |  | 92.805 |  | 152.145 | 95.091 |  | 7.25% |  |
|  |  | L3 | S1 | 0.504 | 0.526 (± 0.016) | 0.814 | 0.846  (± 0.023) | 43.166 | 43.792 (± 0.846) | 69.607 | 43.505 | 44.146  (± 0.858) | 8.57% | 5.218%  (± 0.102%) |
|  |  |  | S2 | 0.534 |  | 0.856 |  | 43.222 |  | 69.718 | 43.574 |  | 8.09% |  |
|  |  |  | S3 | 0.541 |  | 0.869 |  | 44.988 |  | 72.574 | 45.359 |  | 8.32% |  |
| E4 | 3 | L2 | S1 | 1.104 | 0.794 (± 0.258) | 1.891 | 1.362  (± 0.439) | 135.019 | 113.574 (± 17.360) | 231.992 | 144.995 | 121.969  (± 18.639) | 12.23% | 9.560%  (± 1.924%) |
|  |  |  | S2 | 0.473 |  | 0.814 |  | 92.501 |  | 158.948 | 99.343 |  | 19.54% |  |
|  |  |  | S3 | 0.805 |  | 1.380 |  | 113.201 |  | 194.511 | 121.569 |  | 14.07% |  |
|  |  | L4 | S1 | 0.016 | -0.004 (± 0.014) | - | - | - |  | - | - | - | - | - |
|  |  |  | S2 | -0.016 |  | - |  | - |  | - | - |  | - |  |
|  |  |  | S3 | -0.012 |  | - |  | - |  | - | - |  | - |  |
| E5 | 3 | L2 | S1 | 0.822 | 0.549 (± 0.193) | 1.628 | 1.090  (± 0.380) | 58.581 | 55.688 (± 2.520) | 116.224 | 72.640 | 69.055  (± 3.125) | 7.12% | 6.956%  (± 1.807%) |
|  |  |  | S2 | 0.424 |  | 0.842 |  | 52.440 |  | 104.038 | 65.024 |  | 12.38% |  |
|  |  |  | S3 | 0.402 |  | 0.800 |  | 56.042 |  | 111.200 | 69.500 |  | 13.96% |  |
|  |  | L3 | S1 | 1.261 | 1.253 (± 0.068) | 2.456 | 2.438  (± 0.135) | 116.582 | 119.839 (± 16.410) | 226.831 | 141.769 | 145.722  (± 19.953) | 9.24% | 5.951% (± 0.493%) |
|  |  |  | S2 | 1.332 |  | 2.594 |  | 141.367 |  | 275.034 | 171.896 |  | 10.62% |  |
|  |  |  | S3 | 1.165 |  | 2.263 |  | 101.568 |  | 197.602 | 123.501 |  | 8.72% |  |
| E6 | 1 | L1 | S1 | 1.448 | 2.128 (± 0.481) | 2.484 | 3.652  (± 0.826) | 106.702 | 115.754 (± 9.459) | 183.333 | 114.583 | 124.306  (± 10.158) | 7.37% | 3.571%  (± 0.759%) |
|  |  |  | S2 | 2.477 |  | 4.250 |  | 111.752 |  | 192.013 | 120.008 |  | 4.51% |  |
|  |  |  | S3 | 2.459 |  | 4.223 |  | 128.809 |  | 221.324 | 138.328 |  | 5.24% |  |
|  |  | L2 | S1 | 2.527 | 2.49 (± 0.081) | 4.402 | 4.338  (± 0.143) | 127.940 | 138.363 (± 19.320) | 222.898 | 139.311 | 150.662  (± 21.035) | 5.06% | 3.465%  (± 0.399%) |
|  |  |  | S2 | 2.567 |  | 4.471 |  | 165.448 |  | 288.241 | 180.150 |  | 6.45% |  |
|  |  |  | S3 | 2.378 |  | 4.140 |  | 121.702 |  | 212.037 | 132.523 |  | 5.12% |  |
| E7 | 3 | L1 | S1 | 1.213 | 1.300 (± 0.118) | 2.042 | 2.194  (± 0.204) | 138.041 | 167.063 (± 30.400) | 233.179 | 145.737 | 176.378  (± 32.099) | 11.38% | 7.978%  (± 0.715%) |
|  |  |  | S2 | 1.467 |  | 2.484 |  | 209.042 |  | 353.128 | 220.705 |  | 14.25% |  |
|  |  |  | S3 | 1.219 |  | 2.056 |  | 154.105 |  | 260.309 | 162.693 |  | 12.65% |  |
|  |  | L2 | S1 | 0.524 | 0.788 (± 0.253) | 0.911 | 1.375  (± 0.443) | 81.613 | 95.114 (± 17.366) | 142.181 | 88.863 | 103.563  (± 18.906) | 15.56% | 7.910%  (± 1.343%) |
|  |  |  | S2 | 0.712 |  | 1.242 |  | 84.097 |  | 146.515 | 91.572 |  | 11.81% |  |
|  |  |  | S3 | 1.129 |  | 1.973 |  | 119.632 |  | 208.408 | 130.255 |  | 10.60% |  |

| E8 | 3 | L2 | S1 | 1.504 | 1.463 (± 0.046) | 2.939 | 2.857  (± 0.089) | 226.030 | 212.097 (± 9.997) | 441.462 | 275.914 | 258.908  (± 12.202) | 15.03% | 9.066%  (± 0.366%) |
| --- | --- | --- | --- | --- | --- | --- | --- | --- | --- | --- | --- | --- | --- | --- |
|  |  |  | S2 | 1.486 |  | 2.898 |  | 203.053 |  | 396.584 | 247.865 |  | 13.66% |  |
|  |  |  | S3 | 1.398 |  | 2.732 |  | 207.207 |  | 404.713 | 252.945 |  | 14.82% |  |
|  |  | L3 | S1 | 1.842 | 1.811 (± 0.073) | 3.436 | 3.376  (± 0.136) | 223.174 | 232.875 (± 17.166) | 416.374 | 260.234 | 271.544  (± 20.012) | 12.11% | 8.038%  (± 0.399%) |
|  |  |  | S2 | 1.710 |  | 3.188 |  | 218.454 |  | 407.569 | 254.731 |  | 12.78% |  |
|  |  |  | S3 | 1.881 |  | 3.505 |  | 256.997 |  | 479.467 | 299.667 |  | 13.66% |  |
| E9 | 1 | L1 | S1 | 2.040 | 2.034 (± 0.032) | 4.002 | 3.988  (± 0.063) | 101.402 | 104.273 (± 13.361) | 198.817 | 124.260 | 127.782  (± 16.377) | 4.97% | 3.199%  (± 0.362%) |
|  |  |  | S2 | 1.991 |  | 3.905 |  | 89.534 |  | 175.550 | 109.719 |  | 4.50% |  |
|  |  |  | S3 | 2.070 |  | 4.057 |  | 121.882 |  | 238.988 | 149.368 |  | 5.89% |  |
|  |  | L2 | S1 | 1.511 | 1.616 (± 0.128) | 2.870 | 3.073  (± 0.248) | 103.541 | 104.549 (± 14.660) | 196.843 | 123.027 | 124.226  (± 17.418) | 6.85% | 4.031%  (± 0.348%) |
|  |  |  | S2 | 1.797 |  | 3.422 |  | 122.986 |  | 233.813 | 146.133 |  | 6.84% |  |
|  |  |  | S3 | 1.541 |  | 2.926 |  | 87.119 |  | 165.628 | 103.517 |  | 5.65% |  |
| E10 | 3 | L1 | S1 | 2.360 | 2.491 (± 0.127) | 3.616 | 3.818  (± 0.196) | 447.079 | 437.478 (± 9.982) | 685.694 | 428.559 | 419.365  (± 9.563) | 18.95% | 11.012%  (± 0.622%) |
|  |  |  | S2 | 2.663 |  | 4.085 |  | 441.641 |  | 677.373 | 423.358 |  | 16.58% |  |
|  |  |  | S3 | 2.450 |  | 3.754 |  | 423.715 |  | 649.883 | 406.177 |  | 17.30% |  |
|  |  | L2 | S1 | 1.163 | 1.771 (± 0.434) | 1.794 | 2.737  (± 0.673) | 251.712 | 292.063 (± 51.303) | 389.657 | 243.536 | 282.572  (± 49.636) | 21.64% | 10.772%  (± 2.222%) |
|  |  |  | S2 | 2.150 |  | 3.326 |  | 364.458 |  | 564.185 | 352.616 |  | 16.95% |  |
|  |  |  | S3 | 2.000 |  | 3.091 |  | 260.020 |  | 402.505 | 251.565 |  | 13.00% |  |

Table S3: Mine 28 CPDM Data

|  |  |  |  | **RCMD** | | | |  |
| --- | --- | --- | --- | --- | --- | --- | --- | --- |
| **Event** | **MMU** | **Location** | **CPDM Number** | **Mass (mg)** | **Average Mass**  **(mg)** | **Concentration**  **(mg/m^3^)** | **Average Concentration**  **(mg/m^3^)** | **CPDM Errors** |
| E1 | 1 | L1 | 13 | 0.468 | 0.468 | 0.749 | 0.749 |  |
|  |  | L2 | 14 | 0.959 | 0.959 | 1.551 | 1.551 |  |
|  |  | L3 | 12 | 1.886 | 1.886 | 2.926 | 2.926 |  |
|  |  | L4 | 11 | 0.021 | - | - | - |  |
| E2 | 1 | L1 | 12 | 3.965 | 3.965 | 5.777 | 5.777 | HIGH FILTER LOAD |
|  |  | L2 | 11 | 1.856 | 1.856 | 2.748 | 2.748 |  |
|  |  | L3 | - | - | - | - | - |  |
|  |  | L4 | 28 | 0.085 | - | - | - |  |
| E3 | 3 | L1 | 14 | 1.462 | 1.465 | 2.179 | 2.183 |  |
|  |  |  | 27 | 1.467 |  | 2.186 |  |  |
|  |  | L3 | 11 | 0.731 | 0.680 | 1.072 | 0.996 |  |
|  |  |  | 13 | 0.628 |  | 0.921 |  |  |
| E4 | 3 | L2 | 14 | 1.157 | 1.161 | 1.807 | 1.813 |  |
|  |  |  | 27 | 1.164 |  | 1.818 |  |  |
|  |  | L4 | 11 | 0.000 | - | - | - |  |
|  |  |  | 13 | 0.000 |  | - |  |  |
| E5 | 3 | L2 | 14 | 0.823 | 0.825 | 1.484 | 1.487 |  |
|  |  |  | 27 | 0.826 |  | 1.490 |  |  |
|  |  | L3 | 11 | 1.463 | 1.399 | 2.588 | 2.473 |  |
|  |  |  | 13 | 1.334 |  | 2.359 |  |  |
| E6 | 1 | L1 | 11 | 3.085 | 2.965 | 4.819 | 4.631 | MASS OFFSET |
|  |  |  | 13 | 2.845 |  | 4.444 |  | MASS OFFSET |
|  |  | L2 | 14 | 2.702 | 2.644 | 4.279 | 4.188 |  |
|  |  |  | 27 | 2.586 |  | 4.096 |  |  |

| E7 | 3 | L1 | 14 | 1.217 | 1.224 | 1.869 | 1.880 |  |
| --- | --- | --- | --- | --- | --- | --- | --- | --- |
|  |  |  | 27 | 1.231 |  | 1.890 |  |  |
|  |  | L2 | 11 | 1.319 | 1.268 | 2.089 | 2.008 |  |
|  |  |  | 13 | 1.217 |  | 1.927 |  |  |
| E8 | 3 | L2 | 12 | 1.433 | 1.384 | 2.544 | 2.457 |  |
|  |  |  | 19 | 1.335 |  | 2.370 |  |  |
|  |  | L3 | 8 | 1.726 | 1.744 | 2.927 | 2.958 |  |
|  |  |  | 10 | 1.762 |  | 2.988 |  |  |
| E9 | 1 | L1 | 8 | 1.689 | 1.310 | 4.625 | 3.587 | POWER LOW |
|  |  |  | 10 | 0.931 |  | 2.549 |  | POWER LOW |
|  |  | L2 | 12 | 1.844 | 1.785 | 3.187 | 3.085 |  |
|  |  |  | 19 | 1.726 |  | 2.983 |  |  |
| E10 | 3 | L1 | 8 | 2.589 | 2.702 | 3.610 | 3.767 | MASS OFFSET |
|  |  |  | 10 | 2.815 |  | 3.925 |  | MASS OFFSET |
|  |  | L2 | 12 | 2.368 | 2.279 | 3.332 | 3.207 | MASS OFFSET |
|  |  |  | 19 | 2.190 |  | 3.082 |  | MASS OFFSET |

Table S4: NIOSH Method 7603 results for 10 samples.

|  |  |  |  | **RCMD** | | **Quartz** | | |
| --- | --- | --- | --- | --- | --- | --- | --- | --- |
| **Event** | **MMU** | **Location** | **Filter Number** | **MRE Mass (mg)** | **MRE Concentration (mg/m^3^)** | **MRE Mass (mg)** | **MRE Concentration (mg/m^3^)** | **Quartz %** |
| E1 | 1 | L2 | S3 | 1.006 | 1.794 | 0.018 | 0.031 | 1.76% |
| E2 | 1 | L1 | S3 | 2.443 | 3.905 | 0.081 | 0.130 | 3.32% |
| E2 | 1 | L2 | S3 | 0.686 | 1.116 | 0.019 | 0.031 | 2.78% |
| E5 | 3 | L3 | S2 | 1.282 | 2.498 | 0.096 | 0.186 | 7.46% |
| E6 | 1 | L1 | S3 | 2.374 | 4.085 | 0.070 | 0.120 | 2.93% |
| E7 | 3 | L2 | S1 | 0.522 | 0.909 | 0.046 | 0.080 | 8.76% |
| E8 | 3 | L2 | S2 | 1.435 | 2.788 | 0.145 | 0.283 | 10.10% |
| E8 | 3 | L3 | S1 | 1.739 | 3.243 | 0.164 | 0.305 | 9.44% |
| E9 | 1 | L1 | S1 | 1.973 | 3.878 | 0.060 | 0.117 | 3.02% |
| E10 | 3 | L1 | S3 | 2.360 | 3.616 | 0.240 | 0.368 | 10.20% |

Table S5: Summary of data for respirable dust samples lab-generated from Mine 28 dust source materials.

| **MMU** | **Material Type** | **Filter Number** | **Filter Mass (mg)** | **Average Mass (mg)** | **Filter Mass Std. Dev (mg)** | **Quartz Mass (mg)** | **Average Quartz Mass (mg)** | **Quartz Mass Std. Dev (mg)** | **Quartz %** | **Average Quartz %** | **Quartz % Std. Dev** |
| --- | --- | --- | --- | --- | --- | --- | --- | --- | --- | --- | --- |
| 1 | Coal | S1 | 0.774 | 0.737 | 0.060 | 0.006 | 0.006 | 0.004 | 0.78% | 0.88% | 0.69% |
|  |  | S2 | 0.652 |  |  | 0.012 |  |  | 1.77% |  |  |
|  |  | S3 | 0.784 |  |  | 0.001 |  |  | 0.10% |  |  |
|  | Shale (Roof Rock) | S1 | 2.204 | 2.143 | 0.166 | 0.466 | 0.413 | 0.038 | 21.16% | 19.32% | 1.66% |
|  |  | S2 | 1.916 |  |  | 0.377 |  |  | 19.67% |  |  |
|  |  | S3 | 2.31 |  |  | 0.396 |  |  | 17.14% |  |  |
| 3 | Coal | S1 | 1.485 | 1.188 | 0.344 | 0.091 | 0.082 | 0.008 | 6.12% | 7.40% | 1.84% |
|  |  | S2 | 1.373 |  |  | 0.083 |  |  | 6.07% |  |  |
|  |  | S3 | 0.706 |  |  | 0.071 |  |  | 10.00% |  |  |
|  | Bolter Dust (Roof Rock) | S1 | 1.177 | 0.972 | 0.222 | 0.151 | 0.144 | 0.024 | 12.84% | 15.14% | 1.68% |
|  |  | S2 | 0.664 |  |  | 0.111 |  |  | 16.78% |  |  |
|  |  | S3 | 1.075 |  |  | 0.170 |  |  | 15.82% |  |  |
| Entire mine | Rock Dust | S1 | 0.886 | 1.135 | 0.176 | 0.040 | 0.026 | 0.016 | 4.56% | 2.50% | 1.78% |
|  |  | S2 | 1.255 |  |  | 0.034 |  |  | 2.70% |  |  |
|  |  | S3 | 1.263 |  |  | 0.003 |  |  | 0.22% |  |  |
